# The type three secretion system effector protein IpgB1 promotes *Shigella flexneri* cell-to-cell spread through double-membrane vacuole escape

**DOI:** 10.1371/journal.ppat.1010380

**Published:** 2022-02-24

**Authors:** Erin A. Weddle, Volkan K. Köseoğlu, Brittany A. DeVasure, Hervé F. Agaisse

**Affiliations:** Department of Microbiology, Immunology, and Cancer Biology, University of Virginia School of Medicine, Charlottesville, Virginia, United States of America; University of Toronto, CANADA

## Abstract

*S*. *flexneri* is an important human pathogen that causes bacillary dysentery. During infection, *S*. *flexneri* invades colonic epithelial cells, hijacks the host cell cytoskeleton to move in the cytosol of infected cells, and spreads from cell to cell through formation of membrane protrusions that project into adjacent cells and resolve into double membrane vacuoles (DMVs). *S*. *flexneri* cell-to-cell spread requires the integrity of the bacterial type three secretion system (T3SS). However, the exact role of the T3SS effector proteins in the dissemination process remains poorly understood. Here, we investigated the role of the T3SS effector protein IpgB1 in *S*. *flexneri* dissemination. IpgB1 was previously characterized as a guanine nucleotide exchange factor (GEF) that contributes to invasion. In addition to the invasion defect, we showed that the *ipgB1* mutant formed smaller infection foci in HT-29 cells. Complementation of this phenotype required the GEF activity of IpgB1. Using live confocal microscopy, we showed that the *ipgB1* mutant is specifically impaired in DMV escape. Depletion of Rac1, the host cell target of IpgB1 during invasion, as well as pharmacological inhibition of Rac1 signaling, reduced cell-to-cell spread and DMV escape. In a targeted siRNA screen, we uncovered that RhoA depletion restored *ipgB1* cell-to-cell spread and DMV escape, revealing a critical role for the IpgB1-Rac1 axis in antagonizing RhoA-mediated restriction of DMV escape. Using an infant rabbit model of shigellosis, we showed that the *ipgB1* mutant formed fewer and smaller infection foci in the colon of infected animals, which correlated with attenuated symptoms of disease, including epithelial fenestration and bloody diarrhea. Our results demonstrate that, in addition to its role during invasion, IpgB1 modulates Rho family small GTPase signaling to promote cell-to-cell spread, DMV escape, and *S*. *flexneri* pathogenesis.

## Author summary

Bacillary dysentery, caused by the bacterium *Shigella flexneri*, is a severe diarrheal disease that causes more than 200 million cases and over 200,000 deaths worldwide each year. The increasing and alarming presence of *S*. *flexneri* strains resistant to multiple antibiotics generates urgency for increased understanding of how *S*. *flexneri* causes disease. *S*. *flexneri* infects and replicates within human colon cells and spreads throughout the colon, leading to massive tissue destruction and bloody diarrhea. The mechanisms supporting this dissemination process are poorly understood. Here, we characterized one of the bacterial proteins, IpgB1, which allows *S*. *flexneri* to spread throughout the human (host) colon. Upon spreading from one host cell to another, the bacteria are entrapped in host cell membrane compartments termed double membrane vacuoles (DMVs). We found that IpgB1 manipulates host cell signaling proteins to escape DMVs, spread throughout the colon, and cause disease. Our work provides insight into the mechanisms by which *S*. *flexneri* causes disease and suggests novel therapeutic targets.

## Introduction

The human pathogen *Shigella flexneri* is the causative agent of bacillary dysentery, or shigellosis, which causes over 200 million cases and 200,000 deaths annually [1]. The hallmark of *S*. *flexneri* infection is invasion of colonic epithelial cells and intercellular dissemination, leading to destruction of the mucosa and bloody diarrhea [2,3]. The process of intercellular dissemination is critical for *S*. *flexneri* pathogenesis [3], emphasizing the importance of understanding the cellular and molecular mechanisms that support this process.

*S*. *flexneri* encodes a type three secretion system (T3SS) and an arsenal of approximately twenty-five T3SS effector proteins, which manipulate host cell processes during infection. *S*. *flexneri* uses its T3SS to induce its uptake into nonphagocytic epithelial cells and to escape from primary vacuoles following uptake [4]. In the cytosol, *S*. *flexneri* hijacks the host actin cytoskeleton through expression of the autotransporter protein IcsA, leading to bacterial actin-based motility (ABM) [5]. As bacteria collide with cell junctions, they deform the plasma membrane and protrude into neighboring cells. Membrane protrusions are then resolved into vacuole-like protrusions (VLPs) that resolve into double-membrane vacuoles (DMVs) in neighboring cells. The bacteria then escape from DMVs and resume ABM and cell-to-cell spread [6–8]. The T3SS is required for cell-to-cell spread and more recently, the T3SS effector IcsB, an Nε-fatty acyltransferase, was shown to be required specifically for DMV escape during dissemination [8–11]. IcsB is not the only factor facilitating DMV escape, as a proportion of *icsB* bacteria still manage to escape from DMVs, albeit a delayed process [8]. Mutants that are T3SS deficient, completely fail to escape from DMVs, suggesting that additional effectors may be involved in DMV escape. However, the roles of additional effector(s) in the dissemination remain elusive.

As the process of intercellular dissemination relies on bacterial manipulation of host cytoskeleton and membrane, we became interested in T3SS effectors IpgB1 and IpgB2, two bacterial guanine nucleotide exchange factors (GEFs) that regulate Rho family small GTPases [12]. Both IpgB1 and IpgB2 are members of a protein family of bacterial effectors defined by a conserved tryptophan-xxx-glutamic acid (WxxE) motif [13]. Functional and structural insights revealed that IpgB1 and IpgB2 are in fact bacterial GEFs and provided a mechanism for specificity and activation of Rac1 and RhoA by IpgB1 and IpgB2, respectively [14,15]. IpgB1 and IpgB2 were investigated for their roles in *S*. *flexneri* invasion [16–18]. An IpgB1 mutant was less invasive in HeLa cells and overexpression of IpgB1 led to Rac1-dependent membrane ruffling [17,18], while IpgB2 was not required for invasion or spreading in HeLa cells [16,19].

Here, we demonstrate that, in addition to its role during invasion, IpgB1 modulates Rho family small GTPase signaling to promote cell-to-cell spread through DMV escape, with consequences on the severity of *S*. *flexneri* pathogenesis.

## Results

### IpgB1 is required for efficient invasion in HT-29 cells

**Fig 1:**
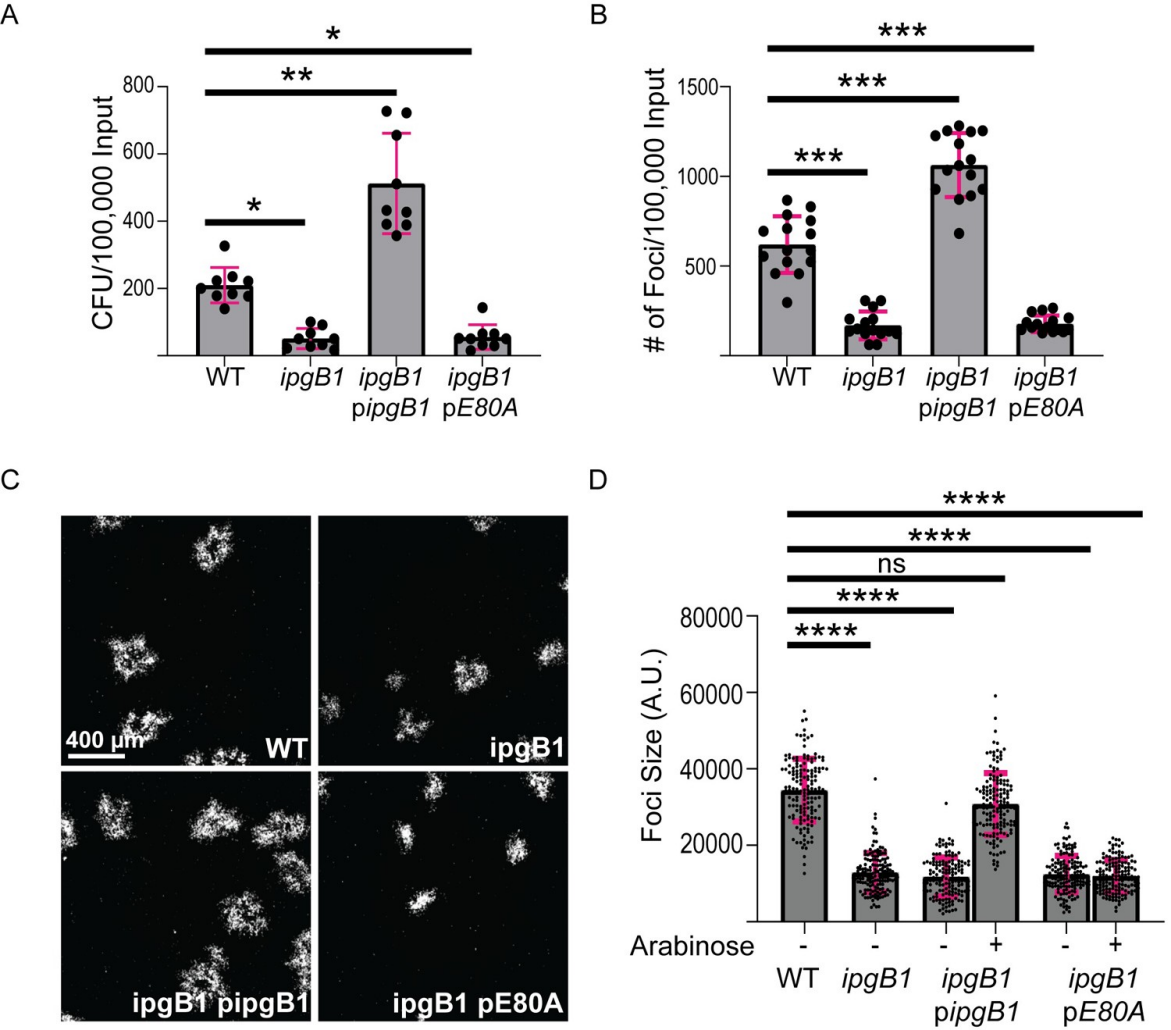
IpgB1 is required for efficient invasion and cell-to-cell spread in HT-29 cells. (A) Intracellular CFUs from HT-29 cells at 2 hr postinfection normalized to inoculum. Three independent biological replicates were performed, each containing three technical replicates. Each point represents one technical replicate and the grey bars represent the mean number of CFU. (B) Number of infection foci formed at 8 hr postinfection normalized to inoculum. Three independent biological replicates were performed, each containing five technical replicates. Each point represents one technical replicate and the grey bars represent the mean number of foci. For *ipgB1* p*ipgB1* and *ipgB1* p*E80A*, arabinose was added at a final concentration of 0.5% during exponential growth and invasion. (C) Representative images showing infection foci formed in HT-29 cells at 16 hr postinfection. The scale bar is 400 μm. (D) Quantification of foci size (area) in arbitrary units at 16 hr postinfection. Three independent biological replicates were performed, each containing at least 50 foci. Each point represents one focus and the grey bars represent the mean foci size. For *ipgB1* p*ipgB1* and *ipgB1* p*E80A*, arabinose was added at a final concentration of 1% at 1 hr postinfection. Error bars represent the standard deviations; one-way ANOVA was performed with Dunnett’s multiple comparisons test; ns, not significant; *, p<0.05; **, p<0.005; ***, p<0.001; ****, p<0.0001.

To investigate the roles of IpgB1 and IpgB2 during *S*. *flexneri* infection, we created mutants lacking either *ipgB1* or *ipgB2* (hereby referred to as *ipgB1* and *ipgB2*) in the *S*. *flexneri* 2457T background by replacing the open reading frame with a kanamycin or chloramphenicol resistance cassette, respectively. We used a gentamicin protection assay in HT-29 cells to determine the roles of IpgB1 and IpgB2 during invasion of polarized colonic epithelial cells. Consistent with previously published results [17], we found that *ipgB1* produced fewer colony forming unit (CFU) at 2 hr postinfection (Fig 1A, WT vs. *ipgB1*) and fewer infection foci at 8 hr postinfection (Fig 1B, WT vs. *ipgB1*) compared to wild type bacteria. Importantly, we were able to restore the invasion defect of the *ipgB1* mutant by expression of IpgB1 *in trans* (Fig 1, *ipgB1* p*ipgB1*). To confirm the role of the previously-established GEF activity of IpgB1 during invasion [14], we generated a catalytically inactive allele of IpgB1 by mutating glutamic acid at residue 80 to alanine (E80A) [14,20]. We found that the E80A allele did not complement the invasion defect of the *ipgB1* mutant (Fig 1, *ipgB1* p*E80A*). Mutants lacking *ipgB2* were fully invasive, producing a similar number of CFUs (S1A Fig, WT vs. *ipgB2*) and infection foci compared to wild type bacteria (S1B Fig, WT vs. *ipgB2*). Taken together, these results show that IpgB1, but not IpgB2, functions as a GEF to facilitate invasion in HT-29 cells.

### IpgB1 is required for efficient dissemination in HT-29 cells

We next sought to determine whether IpgB1 played a role in the dissemination of *S*. *flexneri*. The *ipgB1* mutant formed smaller infection foci compared to wild type bacteria in HT-29 cells at 16 hr postinfection (Fig 1C, WT vs. *ipgB1*). Expression of the wild type, but not the E80A allele of *ipgB1* under the control of an arabinose-inducible promoter restored *ipgB1* spreading, indicating that the GEF activity of IpgB1 was required for efficient dissemination (Fig 1C, *ipgB1* p*ipgB1* vs. *ipgB1* p*E80A*). We quantified the area of the infection foci as a measure of cell-to-cell spread and found that the *ipgB1* mutant had a 62% reduction in foci size compared to wild type bacteria, which was fully rescued by *in trans* expression of wild type IpgB1, but not the E80A allele (Fig 1D). The *ipgB2* mutant formed similarly sized infection foci (S1C Fig, WT vs. *ipgB2*) compared to wild type bacteria, indicating that IpgB2 is dispensable for both invasion and cell-to-cell spread in HT-29 cells. To investigate a potential genetic interaction between IpgB1 and IpgB2, we created a double *ipgB1; ipgB2* mutant and assessed its ability to spread from cell to cell. We found that the *ipgB1; ipgB2* double mutant formed smaller foci compared to wild type and the *ipgB2* mutant, but was not significantly different than the *ipgB1* mutant (S2A Fig). Taken together, these results show that IpgB1 functions as a GEF during cell-to-cell spread in HT-29 cells.

### IpgB1 is required for prompt and efficient DMV escape

**Fig 2:**
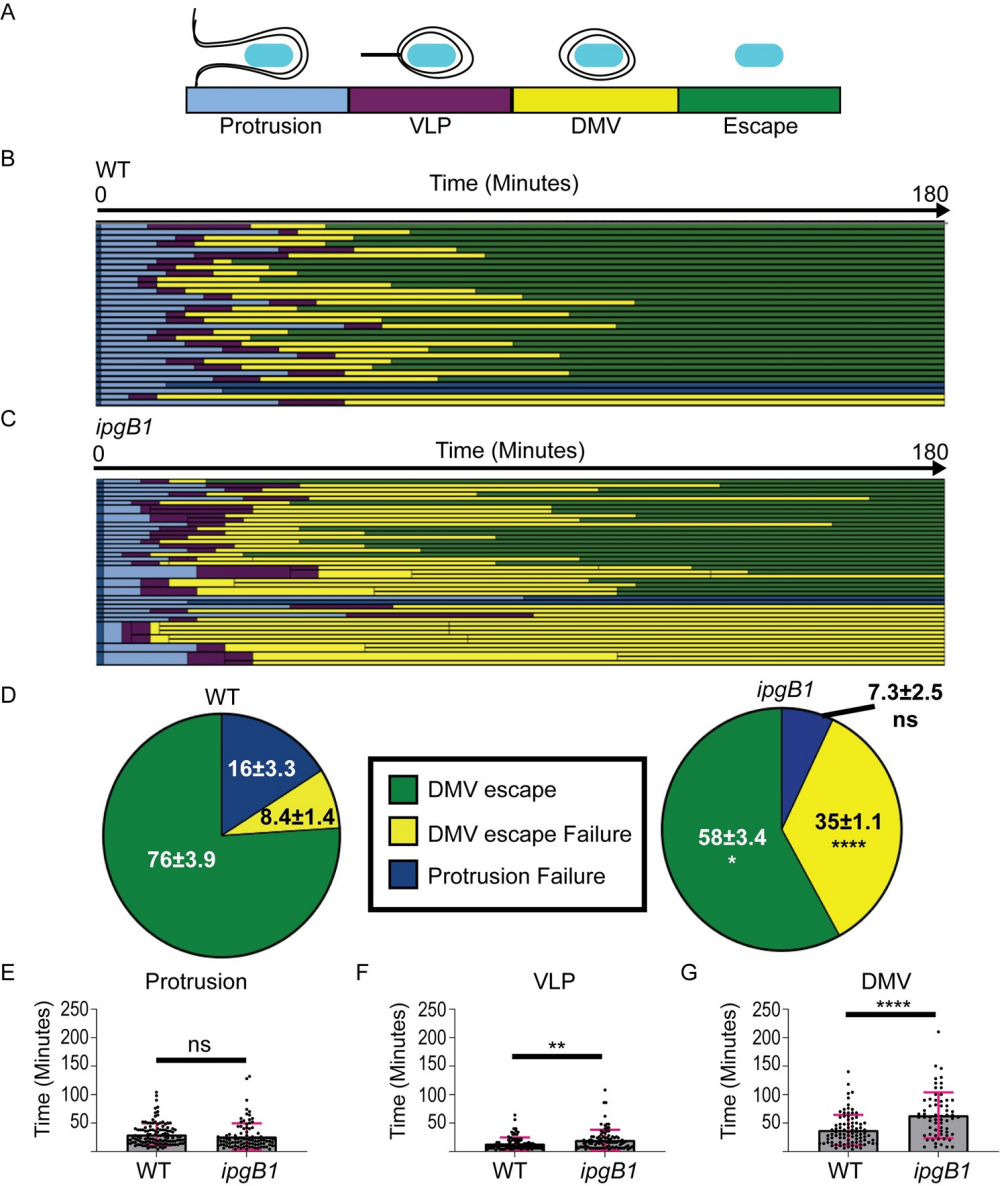
IpgB1 is required for efficient DMV escape. (A) Schematic depicting the stage and corresponding color code of spread progression (B and C) Representative tracking analysis of (B) wild type or (C) *ipgB1* CFP-expressing *S*. *flexneri* in HT-29 cells expressing plasma membrane-targeted YFP. Each bar represents the tracking of a single bacterium over 180 minutes. For *ipgB1*, the thicker bars indicate when bacteria divided and branched during spreading. At least 30 bacteria were tracked for each strain per biological replicate. (D) Graphs showing the proportions of fates of tracked bacteria from 4 biological replicates. Standard deviations of the means are indicated. Two-way ANOVA with Sidak’s multiple comparisons was performed; ns, not significant; *, p<0.05; ****, p<0.0001. Time spent in protrusions (E), VLPs (F), or DMVs (G) is shown for all tracked bacteria. Each dot represents a single bacterium and the grey bars show the mean of all tracked bacteria from four biological replicates. Error bars indicate standard deviation. Unpaired two-tailed t-tests were performed; ns, not significant; **, p<0.005; ****, p<0.0001.

To determine at which stage of intercellular dissemination the *ipgB1* mutant is defective, we employed time-lapse confocal microscopy and tracking of individual bacteria during cell-to-cell spread [8,9,21]. HT-29 cells stably expressing plasma membrane-targeted yellow fluorescent protein (YFP) (mbYFP) were infected with wild type or *ipgB1 S*. *flexneri* expressing cyan fluorescent protein (CFP). Motile bacteria were tracked from the point at which they protruded into a neighboring cell (Fig 2A, light blue, protrusion), through the intermediate stage of vacuole-like protrusion (VLP) (Fig 2A, purple, VLP) [21] to the formation of double membrane vacuoles (DMV) (Fig 2A, yellow, DMV). Subsequent escape from DMVs was marked by the disappearance of mbYFP and the reacquisition of actin-based motility in the adjacent cell (Fig 2A, green, escape) [8]. Tracking of individual bacteria revealed that *ipgB1* bacteria had a decrease in successfully spreading bacteria (Fig 2B and 2C, green bars) and an increase in bacteria remaining trapped in DMVs (Fig 2B and 2C, yellow bars). Quantification of these outcomes revealed that only 58% of the *ipgB1* mutant successfully escaped DMVs, compared to 76% of wild type bacteria (Fig 2D, DMV escape). The *ipgB1* mutant had a significant increase in DMV escape failure (35%) compared to wild type bacteria (8%) (Fig 2D, DMV escape failure). The proportion of bacteria that failed in protrusions was not significantly different between wild type and the *ipgB1* mutant (Fig 2D, protrusion failure). Additionally, the time spent in protrusions was similar between wild type and *ipgB1* bacteria (Fig 2E). Even when spreading was successful, the *ipgB1* mutant spent significantly more time in VLPs (Fig 2F) and in DMVs (Fig 2G) compared to wild type bacteria. These results indicate a role for IpgB1 in prompt and efficient DMV escape. Given our previous work demonstrating that another *S*. *flexneri* T3SS effector, IcsB, is involved in DMV escape [8], we investigated whether IcsB and IpgB1 could be contributing cooperatively to DMV escape. We created a *S*. *flexneri* mutant lacking both *icsB* and *ipgB1* and characterized its capability for cell-to-cell spread. We found that the *icsB; ipgB1* double mutant formed significantly smaller foci than the *icsB* and *ipgB1* single mutants (S2B Fig). This compounded spreading defect of the double mutant suggests that IcsB and IpgB1 play non-redundant roles in cell-to-cell spread.

### The *ipgB1* mutant displays a protrusion branching phenotype

In addition to the canonical dissemination process involving the sequential formation of protrusions, VLPs and DMVs (S3A Fig and S1 Movie and [7–9]), we found that the *ipgB1* mutant had a tendency to divide and resume ABM in VLPs, leading to a protrusion “branching” phenotype (S3B Fig and S2 Movie). We found that about 25% of *ipgB1* mutants underwent protrusion branching, whereas none of the wild type bacteria displayed this phenotype (S3C Fig). To determine if protrusion branching contributed to the cell-to-cell spreading defect of *ipgB1*, we compared the total time spent in membrane (protrusions, VLPs, and DMVs) (S3D Fig), time spent in protrusions and VLPs (S3E Fig), and the time spent in DMVs (S3F Fig) between non-branching and branching *ipgB1* bacteria. We found no significant differences in the timing of non-branching and branching bacteria, indicating that there is no delay in cell-to-cell spread. Furthermore, when we compared the proportions of bacteria that failed to resolve protrusions, remained trapped in DMVs, or successfully escaped DMVs, we found no major differences between non-branching and branching bacteria (S3G Fig). Therefore, we conclude that the branching phenotype observed with the *ipgB1* mutant does not significantly contribute to the observed cell-to-cell spreading defects and that the main phenotype characterized by tracking that contributes to defective *ipgB1* spreading is DMV escape failure.

### Rac1 promotes cell-to-cell spread

**Fig 3:**
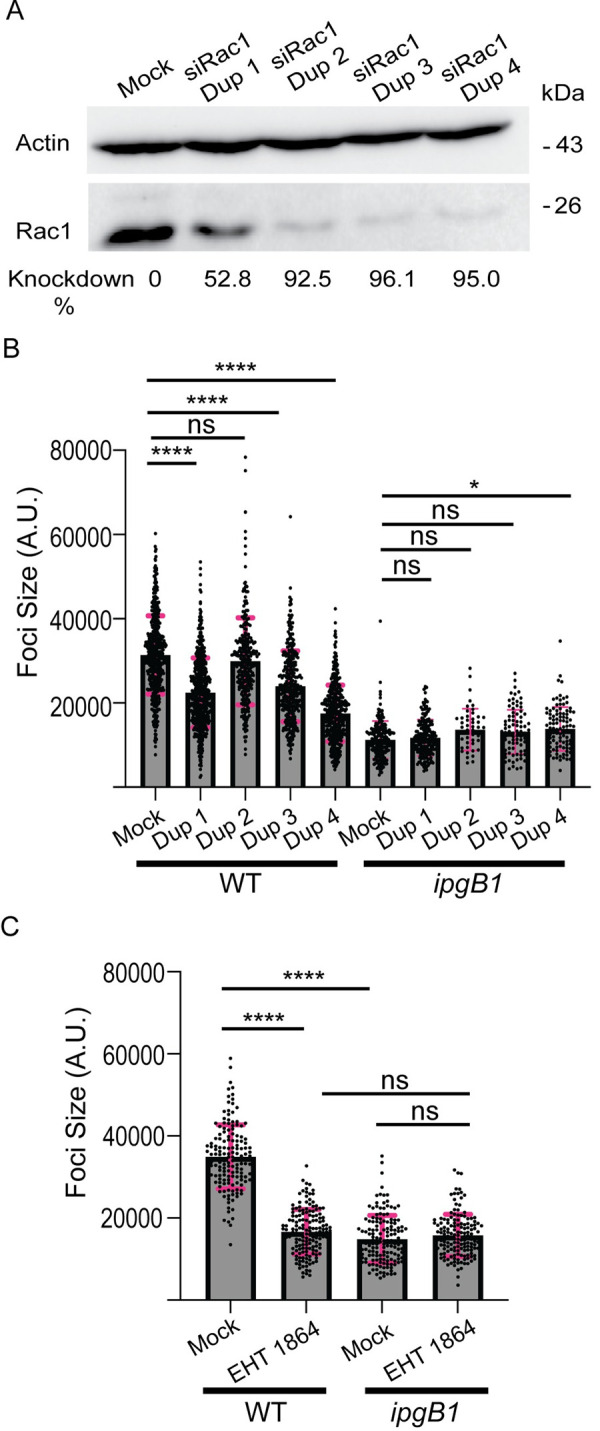
Rac1 signaling is required for efficient cell-to-cell spread. (A) Western blot showing knockdown efficiency of four siRNA duplexes targeting Rac1. Rac1 (bottom panel) was normalized to loading control (actin, top panel) and knockdown efficiency of was calculated relative to mock treated cells. (B) Quantification of foci size at 16 hr postinfection of wild type or *ipgB1* bacteria in mock treated cells or upon Rac1 depletion with four siRNA duplexes. (C) Quantification of wild type or *ipgB1* foci size at 16 hr postinfection in either cells treated with DMSO control or with 20 μM EHT 1864. For (B and C), at least three independent biological replicates were performed, each containing at least 50 foci. Each point represents one focus and the grey bars represent the mean foci size. Error bars represent the standard deviations; one-way ANOVA was performed with Dunnett’s multiple comparisons test; ns, not significant; *, p<0.05; ****, p<0.0001.

Given that IpgB1 was previously found to function as a GEF with specificity for the small GTPase Rac1 [14], we decided to investigate the contribution of Rac1 to cell-to-cell spread. We depleted HT-29 cells of Rac1 using four independent siRNA duplexes and assessed the effect on foci size. We found that three out of four of the tested siRNA duplexes significantly decreased the spreading of wild type *S*. *flexneri* (Fig 3A and 3B). Additionally, we found that pharmacological inhibition of Rac1 using the inhibitor EHT 1864 decreased wild type spreading to a foci size similar to the size of the foci formed by *ipgB1* (Fig 3C). Importantly, we did not observe any decrease in *ipgB1* foci size upon genetic (Fig 3B) or chemical (Fig 3C) interference with Rac1 activity. These results reveal that Rac1 supports cell-to-cell spread through a mechanism that depends on IpgB1 expression.

### Rac1 promotes DMV escape

We next determined whether Rac1 depletion phenocopied the DMV escape defect observed in cells infected with the *ipgB1* mutant. To this end, we used live confocal microscopy to track the spreading of wild type *S*. *flexneri* in either mock-treated (S4A Fig) or Rac1-depleted (S4B Fig) HT-29 cells expressing mbYFP. We found that depletion of Rac1 led to an increase in the proportion of wild type bacteria that remained trapped in DMVs (S4A and S4B Fig, yellow bars). Unexpectedly, we also found an increase in protrusion resolution failure upon Rac1 depletion (S4A and S4B Fig, dark blue bars). Given that we did not observe this increase in protrusion failure with the *ipgB1* mutant, we reasoned that this could be the result of an additional, IpgB1-independent role of Rac1 in cell-to-cell spread, such as the specification of proper cell-cell contacts [22], which is required for efficient protrusion resolution into vacuoles in polarized cells [21]. Quantification of our tracking results revealed that only 26% of bacteria successfully escaped DMVs in Rac1-depleted cells, compared to 74% of bacteria in mock-treated cells (S4C Fig, DMV escape). Rac1 depletion significantly increased DMV escape failure (38%) compared to mock-treated cells (12%) (S4C Fig, DMV escape failure). As described above, the proportion of bacteria that failed to resolve protrusions was significantly higher in Rac1-depleted cells compared to mock-treated cells (S4C Fig, protrusion failure), but the time spent in protrusions (S4D Fig), VLPs (S4E Fig) or DMVs (S4F Fig) did not significantly differ.

Taken together, these data indicate that genetic or chemical interference with Rac1 phenocopies the *ipgB1* mutant in terms of cell-to-cell spread and DMV escape.

### RhoA depletion rescues the *ipgB1* cell-to-cell spreading defect

**Fig 4:**
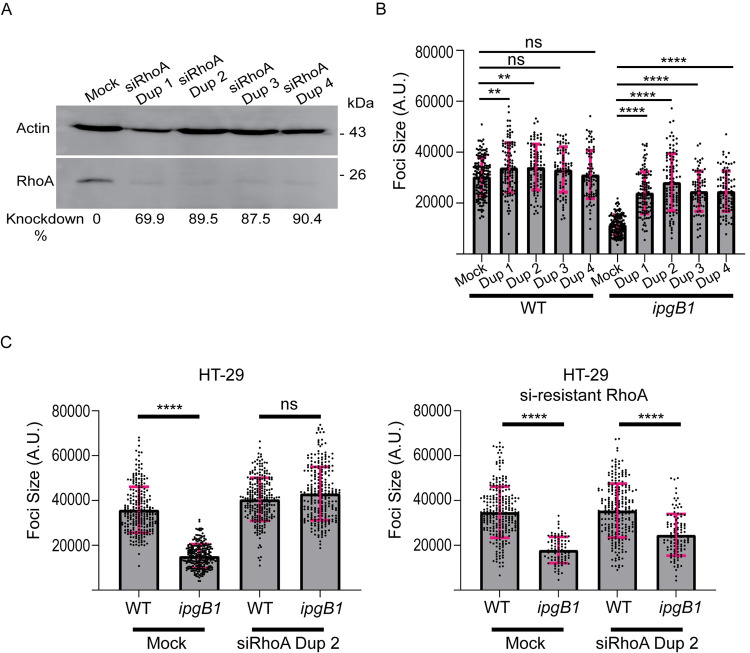
RhoA depletion restores *ipgB1* cell-to-cell spread. (A) Western blot showing knockdown efficiency of four siRNA duplexes targeting RhoA. RhoA (bottom panel) was normalized to loading control (actin, top panel) and knockdown efficiency was calculated relative to mock treated cells. (B) Quantification of wild type or *ipgB1* foci size at 16 hr postinfection in mock treated cells or cells transfected with four RhoA-targeting siRNA duplexes. (C) Quantification of wild type or *ipgB1* foci size at 16 hr postinfection in HT-29 cells (left graph, HT-29) or HT-29 cells stably expressing siRNA duplex 2-resistant mCherry RhoA (right graph, HT-29 si-resistant RhoA) under mock-treated conditions or upon knockdown of RhoA with siRNA duplex 2. For (B and C), at least three independent biological replicates were performed, each containing at least 50 foci. Each point represents one focus and the grey bars represent the mean foci size. Error bars represent the standard deviations; one-way ANOVA was performed with Dunnett’s multiple comparisons test; ns, not significant; **, p<0.005; ****, p<0.0001.

To identify any potentially novel relationships between IpgB1 and Rho family small GTPases that could be contributing to *S*. *flexneri* cell-to-cell spread downstream of IpgB1, we performed a targeted siRNA screen of common Rho family members and related proteins (S2 Table). HT-29 cells were transfected with four single siRNA duplexes targeting each gene of interest and were then infected with either wild type bacteria or the *ipgB1* mutant. We considered genes as potential “hits” if knockdown with at least two of the four duplexes influenced the size of the infection foci (spreading) by 1.5 or more standard deviations in at least three of the four replicates that we performed. Our siRNA screen results revealed that knockdown of RhoA led to an increase in *ipgB1*, but not wild type, cell-to-cell spread (S2 Table). We confirmed the efficiency of knockdown of RhoA with four siRNA duplexes using a RhoA antibody and found that all four duplexes resulted in 70–90% depletion of RhoA (Fig 4A). To confirm our screening results, we measured the size of wild type or *ipgB1* infection foci formed in mock-treated or RhoA-depleted HT-29 cells at 16 hrs postinfection. All four duplexes significantly restored *ipgB1* cell-to-cell spread to near wild type levels (Fig 4B, *ipgB1*). Two duplexes caused a modest increase in wild type cell-to-cell spread (Fig 4B, WT). To determine whether the restoration of *ipgB1* cell-to-cell spread was specifically due to RhoA depletion, we created an HT-29 cell line that stably overexpressed an allele of RhoA resistant to siRNA treatment with siRNA duplex 2 targeting RhoA. In HT-29 cells, we found that the *ipgB1* mutant displayed attenuated spreading compared to wild type (Fig 4C, HT-29, Mock) that was fully restored upon depletion of RhoA by siRNA duplex 2 (Fig 4C, HT-29, siRhoA Dup 2). In HT-29 cells overexpressing siRNA-resistant RhoA (HT-29 si-resistant RhoA), we found that *ipgB1* was still defective in cell-to-cell spread compared to wild type *S*. *flexneri* (Fig 4C, HT-29 si-resistant RhoA, Mock). This defect was however no longer restored upon treatment with siRNA duplex 2 (Fig 4C, HT-29 si-resistant RhoA, siRhoA Dup 2), indicating that overexpression of siRNA-resistant RhoA prevented restoration of *ipgB1* cell-to-cell spread by RhoA knockdown. These results indicate that the restoration of *ipgB1* cell-to-cell spread is specifically due to RhoA depletion.

### RhoA restricts DMV escape

**Fig 5:**
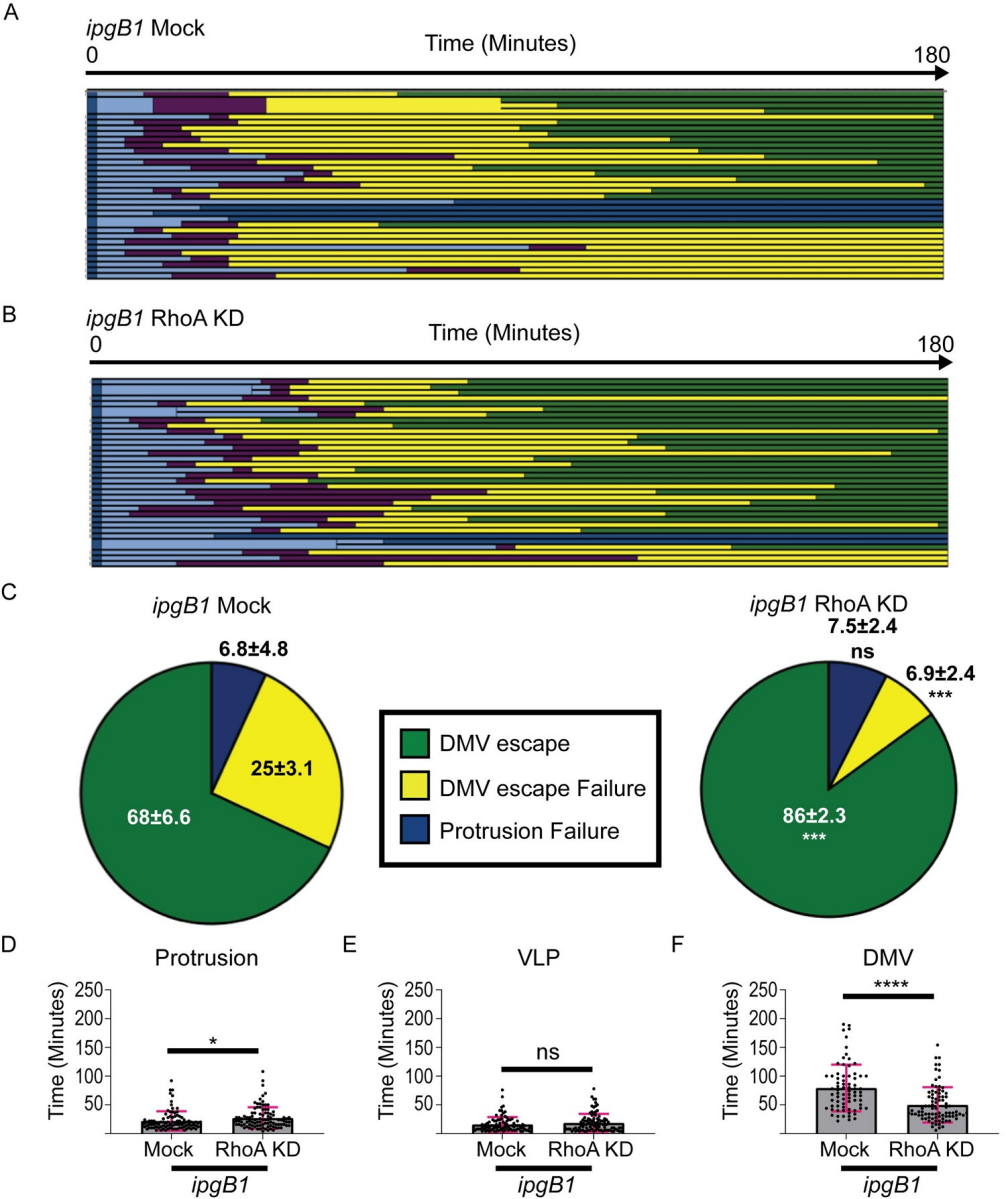
RhoA depletion restores *ipgB1* DMV escape. (A and B) Representative tracking analysis of CFP-expressing *ipgB1* bacteria in (A) mock-treated or (B) RhoA duplex 2-depleted HT-29 cells expressing plasma membrane-targeted YFP. Each bar represents the tracking of a single bacterium over 180 minutes. Thicker bars indicate instances when bacteria divided and branched during spreading. At least 30 bacteria were tracked for condition per biological replicate. (C) Graphs showing the proportions of fates of tracked bacteria from three biological replicates. Standard deviations of the means are indicated. Two-way ANOVA with Sidak’s multiple comparisons was performed; ns, not significant; ***, p<0.001. Time spent in protrusions (D), VLPs (E), or DMVs (F) is shown for all tracked bacteria. Each dot represents a single bacterium and the grey bars show the mean of all tracked bacteria from three biological replicates. Error bars indicate standard deviation. Unpaired two-tailed t-tests were performed; ns, not significant; *, p<0.05; ****, p<0.0001.

We next investigated the mechanism of *ipgB1* spreading restoration upon RhoA knockdown by tracking of *ipgB1* cell-to-cell spread in either mock-treated (Fig 5A) or RhoA-depleted (Fig 5B) HT-29 cells. Consistent with our previous results, we found that a substantial proportion (~25%) of *ipgB1* bacteria remained trapped in DMVs (Fig 5C, *ipgB1* Mock, yellow) and this proportion was significantly decreased (~7%) upon depletion of RhoA (Fig 5C, *ipgB1* RhoA KD, yellow). Consistently, the proportion of *ipgB1* bacteria that successfully spread to neighboring cells was significantly increased from 68% to 86% upon RhoA knockdown (Fig 5C, green). We did not observe a difference in the proportion of bacteria that failed to transition from protrusions to VLPs (Fig 5C, blue). We found a very small, but statistically significant increase in the time that *ipgB1* bacteria spent in protrusions upon RhoA knockdown (Fig 5D). There was no difference in time spent in VLPs between mock-treated and RhoA-depleted conditions (Fig 5E). Finally, we found that *ipgB1* bacteria spent significantly less time in DMVs in RhoA-depleted cells compared to mock- treated cells (Fig 5F). These data indicate that siRNA-mediated depletion of RhoA rescued *ipgB1* cell-to-cell spread to wild type levels by restoring prompt and efficient DMV escape. Taken together, our results indicate that IpgB1 expression is required to antagonize the restriction on DMV escape mediated by RhoA.

### IpgB1 enhances Rac1 recruitment and antagonizes RhoA recruitment to DMVs

**Fig 6:**
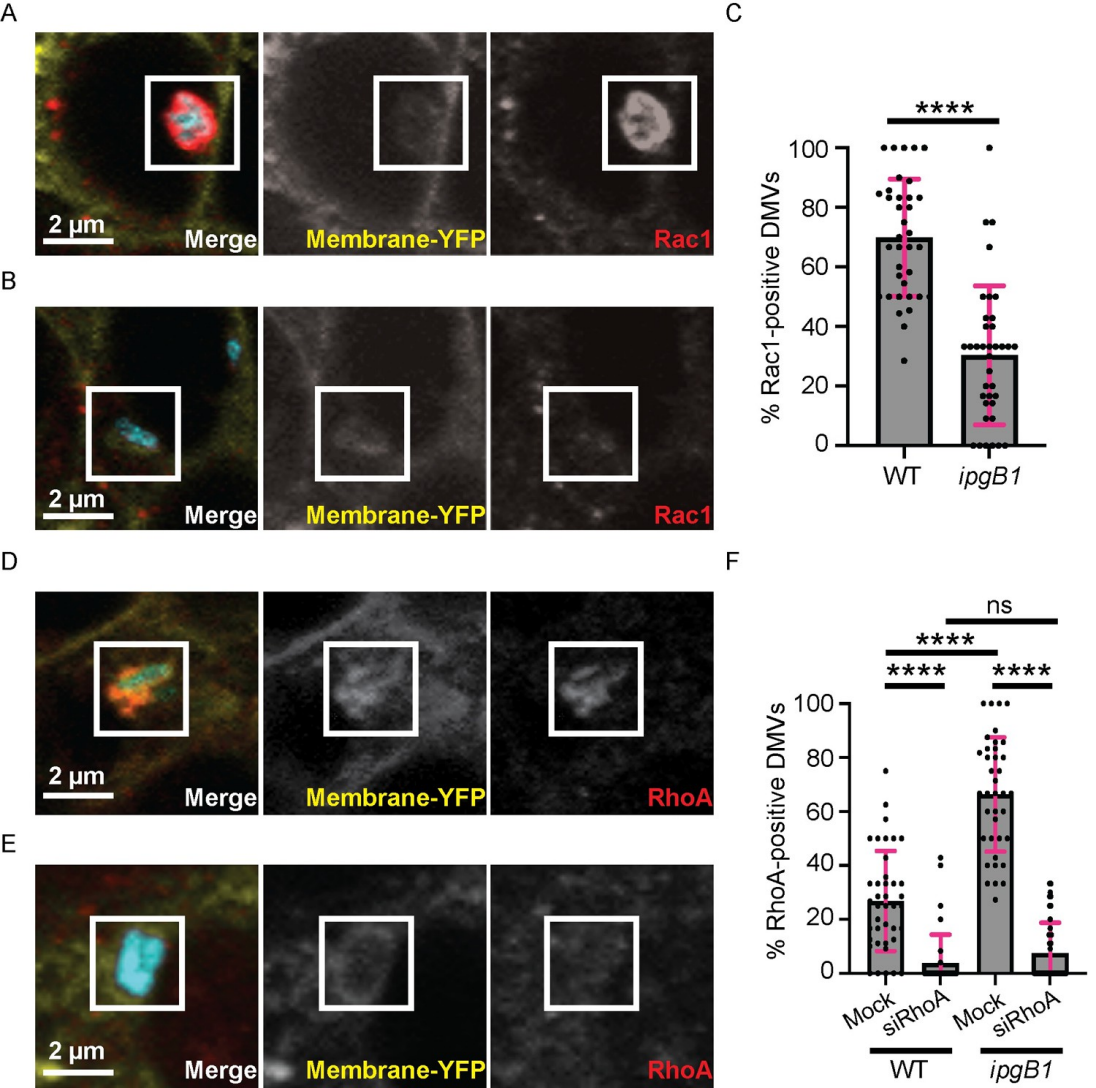
IpgB1 enhances Rac1 and antagonizes RhoA recruitment to DMVs. (A and B) Confocal images of HT-29 cells stably expressing plasma membrane-targeted YFP and infected for 5 hr with CFP-expressing *S*. *flexneri* and stained using an anti-Rac1 antibody followed by AlexFluor 594-conjugated anti-mouse secondary antibody. Merged images are shown on the left, single YFP channel in middle, and single mCherry channel on the right. Scale bar, 2 μm. (A) Representative example of a DMV that is positive for Rac1 recruitment. (B) Representative example of a DMV that is negative for Rac1 recruitment. (C) Quantification of the percentage of wild type or *ipgB1* DMVs per foci associated with Rac1 HT-29 cells. Each dot represents the percent Rac1-positive DMVs in an infection focus and the grey bars show the mean percentage of Rac1-positive DMVs from all foci measured in three biological replicates. (D and E) Confocal images of HT-29 cells stably expressing plasma membrane-targeted YFP and infected for 5 hr with CFP-expressing *S*. *flexneri* and stained using an anti-RhoA antibody followed by AlexFluor 594-conjugated anti-mouse secondary antibody. Merged images are shown on the left, single YFP channel in middle, and single mCherry channel on the right. Scale bar, 2 μm. (D) Representative example of a DMV that is positive for RhoA recruitment. (E) Representative example of a DMV that is negative for RhoA recruitment. (F) Quantification of the percentage of wild type or *ipgB1* DMVs per foci associated with RhoA in either mock-treated or RhoA duplex 2-depleted HT-29 cells. Each dot represents the percent RhoA-positive DMVs in a focus and the grey bars show the mean percentage of RhoA-positive DMVs from all foci measured in three biological replicates. At least twelve foci per condition were analyzed in each biological replicate. Error bars indicate standard deviation. One-way ANOVA with Tukey’s multiple comparisons; ns, not significant; ****, p<0.0001.

Given the IpgB1-dependent role of Rac1 in cell-to-cell spread, we tested whether IpgB1 enhanced the recruitment of Rac1 to DMVs. We infected mbYFP-expressing HT-29 cells with CFP-expressing wild type or *ipgB1 S*. *flexneri* for 5 hrs and then imaged endogenous Rac1 recruitment by immunofluorescence microscopy. We observed DMVs displaying substantial recruitment of Rac1 (Fig 6A) or lacking Rac1 recruitment (Fig 6B). Around 70% of wild type DMVs were positive for Rac1, while only 30% of *ipgB1* DMVs recruited Rac1 (Fig 6C), demonstrating that IpgB1 increases Rac1 recruitment to DMVs.

Because RhoA depletion restored DMV escape and cell-to-cell spread of the *ipgB1* mutant, we wondered whether IpgB1 expression was antagonizing RhoA recruitment to DMVs. To investigate this, we infected mbYFP-expressing HT-29 cells with CFP-expressing wild type or *ipgB1 S*. *flexneri* for 5 hrs and then imaged endogenous RhoA by immunofluorescence microscopy. We observed examples of massive recruitment of RhoA to DMVs (Fig 6D) as well as DMVs that were devoid of RhoA recruitment (Fig 6E). About 25% of wild type DMVs were RhoA-positive (Fig 6F, WT, mock). Strikingly, we observed a dramatic increase in the percentage of RhoA-positive DMVs in cells infected with the *ipgB1* mutant (Fig 6F, *ipgB1*, mock). RhoA knockdown significantly decreased the percentage of RhoA-positive DMVs observed in cells infected with the *ipgB1* mutant to wild type levels (Fig 6F, siRhoA). These results are in agreement with the notion that IpgB1 supports DMV escape by antagonizing RhoA recruitment to DMVs.

Additionally, we investigated the interplay between Rac1 and RhoA recruitment on DMVs by probing the effect of Rac1 depletion on the recruitment of RhoA to DMVs. We found that Rac1 depletion led to an increase in RhoA-positive DMVs in cells infected with wild type bacteria (S5A Fig, WT). Consistent with IpgB1 and Rac1 functioning in the same pathway, we found no significant difference in the percentage of RhoA-positive DMVs upon Rac1 depletion in cells infected with the *ipgB1* mutant (S5A Fig, *ipgB1*). We also examined the proportion of Rac1-positive DMVs upon RhoA depletion. There was no difference in the percentage of Rac1-positive DMVs in RhoA-depleted cells infected with wild type bacteria (S5B Fig, WT). As expected, the percentage of Rac1-positive DMVs was decreased in cells infected with the *ipgB1* mutant (S5B Fig, *ipgB1*). However, upon RhoA depletion, the percentage of Rac1-positive DMVs was restored to wild type level (S5B Fig, *ipgB1*). These data further support the notion that IpgB1 modulates Rac1 and RhoA recruitment to DMVs.

### RhoA does not promote actin polymerization around DMVs

One of the most well studied functions of RhoA in cellular biology is regulation of the actin cytoskeleton. Several vacuole-residing pathogens stimulate the formation of actin structures around their vacuoles. Furthermore, the polymerization of actin around pathogen-containing vacuoles (actin cage) has been shown to be regulated by RhoA modulation [23–26]. To determine whether the formation of RhoA-mediated actin cage could interfere with DMV escape, we investigated the percentage of *S*. *flexneri* DMVs that were surrounded by F-actin during infection of mbYFP-expressing HT-29 cells infected with CFP-expressing bacteria. Upon quantification of actin-positive (S6A Fig) and actin-negative (S6B Fig) DMVs, we found no difference between wild type and the *ipgB1* mutant (S6C Fig). These data suggest that the DMV escape defect of *ipgB1*, which is restored by RhoA depletion, is probably not due to differences in actin cage formation around DMVs.

### IpgB1 contributes to *S*. *flexneri* invasion and cell-to-cell spread *in vivo*

**Fig 7:**
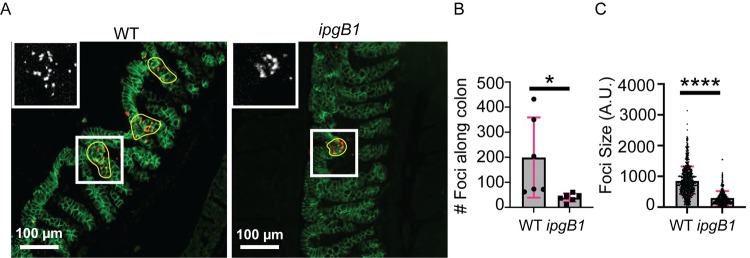
IpgB1 contributes to *S*. *flexneri* invasion and cell-to-cell spread *in vivo*. (A) Representative images of colon sections infected with wild type or *ipgB1 S*. *flexneri* at 8 hr postinfection. Yellow lines delineate individual infection focus and demonstrate how foci size is quantified. Insets in the top left show a zoom-in of the area outlined by the white box and displays single channel image corresponding to *S*. *flexneri* in an infection focus. E-cadherin, green; *S*. *flexneri*, red. Scale bar, 100 μm (B) Graph depicting total number of foci measured along entire colon at 4 hr postinfection. Each dot represents one animal and grey bars show average of six biological replicates. Error bars indicate standard deviation. Unpaired two-tailed t-test; *, p<0.05 (C) Graph depicting foci size in infected colons at 8 hr postinfection. Each dot represents one focus and grey bars show average foci size across six biological replicates. Error bars indicate standard deviation. Unpaired two-tailed t-test; ****, p<0.0001.

In order to correlate our findings in HT-29 cells to *in vivo* infection, we used the recently developed infant rabbit model of shigellosis [3]. We infected animals with wild type or *ipgB1* bacteria and harvested colons 4 hr or 8 hr postinfection to measure invasion (foci number) and cell-to-cell spread (foci size), respectively. Colons were immunostained with antibodies against E-cadherin to visualize epithelial cells (Fig 7A, green) and *S*. *flexneri* (Fig 7A, red), and imaged using epifluorescence microscopy. To measure invasion, we enumerated foci along the entire colon. We found the colons infected with the *ipgB1* mutant contained significantly fewer foci than those infected with wild type bacteria (Fig 7B), indicating that IpgB1 is required for efficient invasion of colonic epithelial cells *in vivo*. As with our foci size experiments in HT-29 cells, we used the Metamorph software to delineate the borders of individual foci along the colons (Fig 7A, yellow lines) and derive the corresponding areas as a measure of cell-to-cell spread efficiency. Infection foci formed in animals infected by the *ipgB1* mutant were significantly smaller than those infected by wild type bacteria (Fig 7C), demonstrating a role for IpgB1 in cell-to-cell spread *in vivo*. Taken together, these results validate our work in HT-29 cells by unveiling a role for IpgB1 during invasion and intracellular dissemination *in vivo*.

### IpgB1 contributes to *S*. *flexneri* pathogenesis

**Fig 8:**
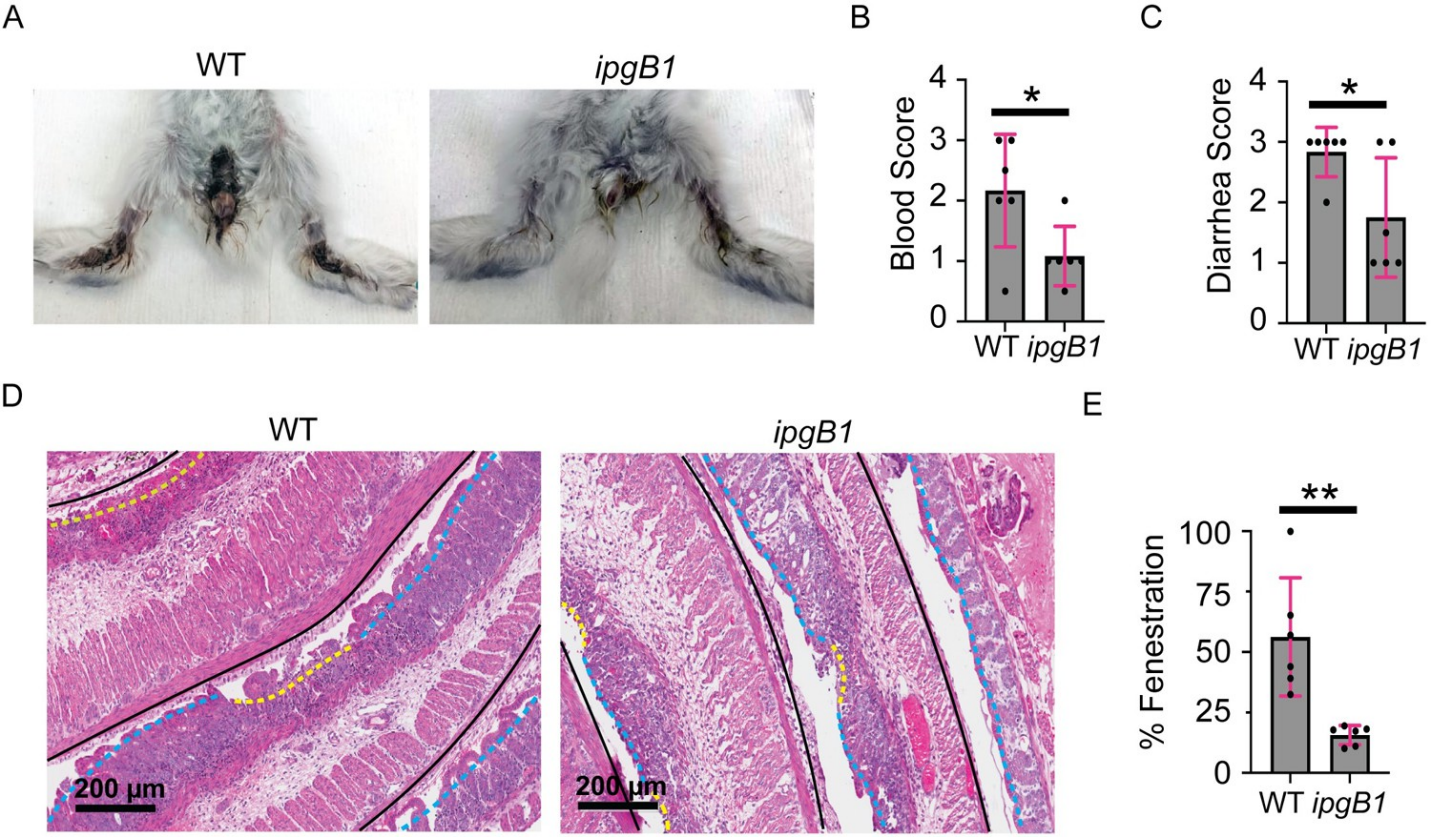
IpgB1 contributes to *S*. *flexneri* pathogenesis. (A) Representative images of animals infected with wild type or *ipgB1 S*.*flexneri* 24 hr postinfection. (B and C) Histopathology scores for blood in stool (B) or diarrhea (C) of animals at 24 hr postinfection with wild type or *ipgB1* strains. Each dot represents one animal and grey bars show average of six biological replicates. Error bars indicate standard deviation. Unpaired two-tailed t-test; *, p<0.05 (D) Representative images of hematoxylin- and eosin-stained colon sections from animals infected with wild type or *ipgB1* at 24 hr postinfection. Black lines delineate the colon. Blue dashed lines indicate areas of intact epithelium and yellow dashed lines indicate areas with epithelial fenestration. Scale bar, 200 μm. (E) Graph depicting the percentage of epithelial fenestration along entire colon. Each dot represents one animal and grey bars show average of six biological replicates. Error bars indicate standard deviation. Unpaired two-tailed t-test; **, p<0.005.

We have recently demonstrated the critical importance of cell-to-cell spread in *S*. *flexneri* pathogenesis [3]. To determine how the defects observed with the *ipgB1* mutant would relate to pathogenesis, we infected infant rabbits with either wild type bacteria or the *ipgB1* mutant. At 24 hr postinfection, animals infected with wild type bacteria displayed characteristic bloody diarrhea, while animals infected with *ipgB1* bacteria had attenuated symptoms (Fig 8A). Scoring for severity of symptoms for blood in stool (Fig 8B) and severity of diarrhea (Fig 8C) revealed that infection with the *ipgB1* mutant resulted in less blood and less severe diarrhea compared to infection with wild type. The animals were dissected at 24 hr postinfection and the colons were harvested and hematoxylin- and eosin-stained to score histopathology. For each infected animal, the entire colon was measured and scored for intact epithelium (Fig 8D, blue dashed lines) or fenestrated epithelium (Fig 8D, yellow dashed lines). The % fenestration along entire colon was calculated by dividing the length of colon with fenestrated epithelium by the total length of the colon. Colons from animals infected with the *ipgB1* mutant had significantly less epithelial fenestration compared to colons from infected with wild type bacteria (Fig 8E). These data indicate that IpgB1 significantly contributes to epithelial fenestration and bloody diarrhea during *in vivo* infection.

## Discussion

**Fig 9:**
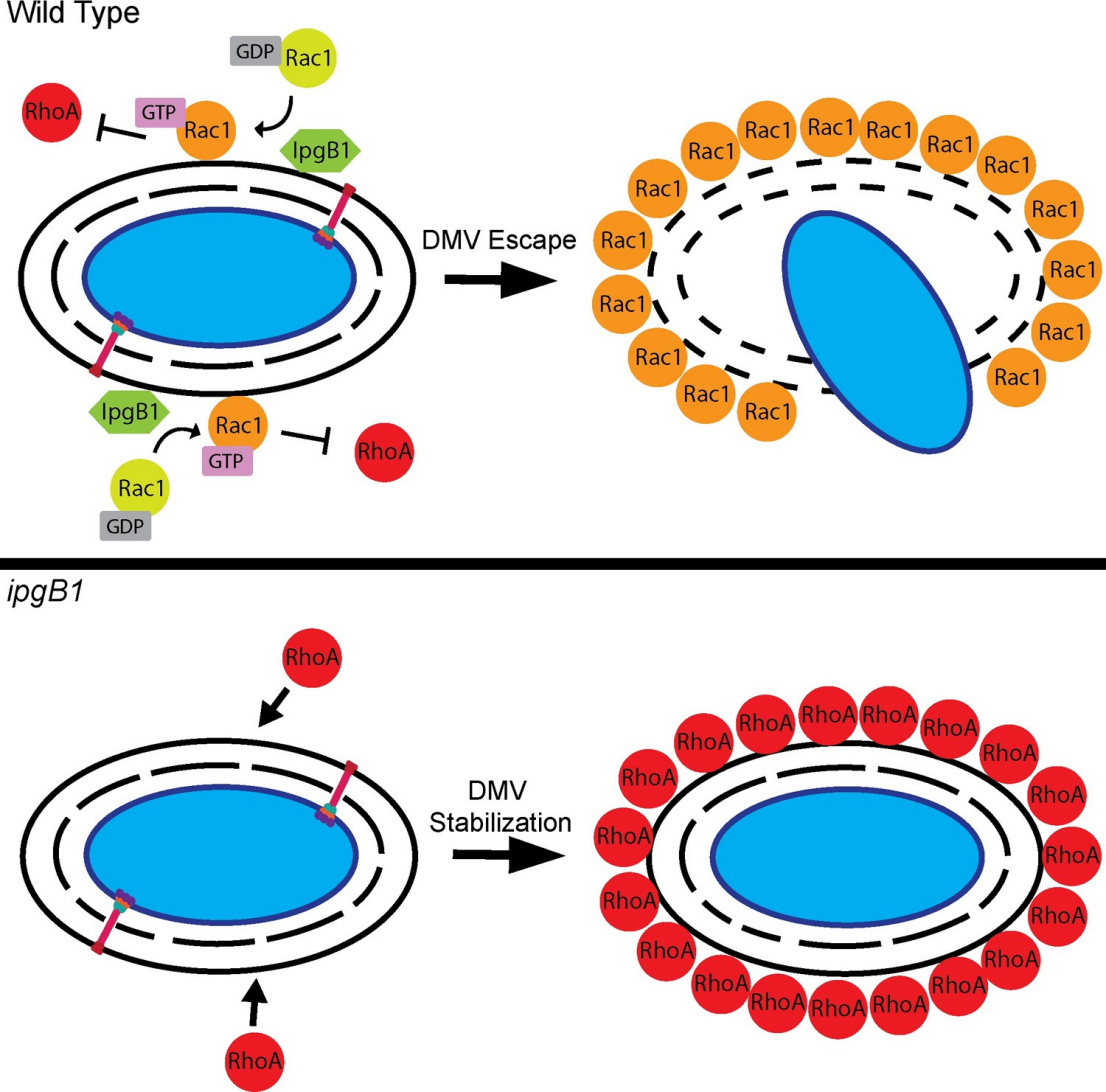
Model for the role of IpgB1 in DMV escape during *S*.*flexneri* cell-to-cell spread. In wild type *S*. *flexneri*, the type three secretion bacterial GEF, IpgB1, recruits Rac1 to DMVs. The IpgB1-Rac1 pathway antagonizes RhoA recruitment to the DMVs to promote DMV escape. The *ipgB1* mutant has diminished ability to recruit Rac1 to the DMV, which allows for the accumulation of RhoA and initiation of RhoA-mediated DMV stabilization, which impedes DMV escape.

*S*. *flexneri* invasion and ABM have been well studied over several decades, but how the bacteria achieve intercellular dissemination (cell-to-cell spread) is still poorly understood. For instance, the *Shigella* GEFs IpgB1 and IpgB2 are known to play a role during invasion of epithelial cells ([13] and Figs 1A and 1B and S1A and S1B). However, their potential role during later stages of infection is unclear. Here we characterized the role of IpgB1 and IpgB2 during *S*. *flexneri* dissemination in epithelial cells. We found that, similar to previously published results [19], IpgB2 is not required for cell-to-cell spread. However, we report a novel role for IpgB1 during cell-to-cell spread through DMV escape. As discussed below and summarized in Fig 9, our results suggest a model of DMV escape in which IpgB1 and Rac1 facilitate DMV escape by antagonizing the recruitment of RhoA to DMVs.

### How does IpgB1 antagonize the recruitment of RhoA?

Our results show that IpgB1 mediates *S*. *flexneri* dissemination by modulating host cell Rho GTPases. IpgB1 was found to have GEF specificity for Rac1 *in vitro*, with little activity on Cdc42, and no activity on RhoA [14]. Consistently, we found that the role of IpgB1 in cell-to-cell spread relied on its GEF activity. Moreover, similar to IpgB1, we found that Rac1 supported cell-to-cell spread through DMV escape. Our results are therefore in agreement with the notion that, similar to the invasion process, IpgB1 functions as a positive regulator of Rac1 during *S*. *flexneri* dissemination. Interestingly, we uncovered that depletion of RhoA restored *ipgB1* cell-to-cell spread and DMV escape to wild type levels. In addition, we found that IpgB1 expression antagonized the recruitment of RhoA to DMVs. Altogether these results suggest that the role of IpgB1 during cell-to-cell spread is to activate Rac1 to antagonize the recruitment of RhoA (Fig 9, Wild Type). The spatial and temporal antagonism between Rac1 and RhoA has been well documented in the cell biology field [27–32]. During cell migration, Rac1 and RhoA are found predominantly at the leading and trailing edges of the cell, respectively [31]. Recent studies also elegantly demonstrated the spatiotemporal oscillating waves of Rac1 and RhoA activity coordinate cell protrusion and edge dynamics [28,33]. Rac1/RhoA antagonism has also been found to drive cell shape, invagination, and heterogeneity during epithelial cell morphogenesis [29,30]. Mechanistically, Rac1 counteracts RhoA through activation or inactivation of signaling components displaying RhoGAP or RhoGEF activity, respectively [34,35]. The exact mechanism supporting Rac1/RhoA antagonism in the context of cell-to-cell spread remains to be determined. Our results however reveal that *S*. *flexneri* has evolved the IpgB1-dependent ability to take advantage of the cell intrinsic antagonism between Rac1 and RhoA in order to block RhoA-mediated restriction of DMV escape (Fig 9, *ipgB1*).

### How does RhoA restrict DMV escape?

#### The actin cytoskeleton hypothesis

The most studied role of Rho family GTPases, including RhoA, is regulation of the actin cytoskeleton. RhoA-dependent actin polymerization has been implicated in the stabilization of the pathogen-containing vacuole during *Chlamydia trachomatis* infection [25]. *C*. *trachomatis* induces the recruitment of RhoA and the formation of actin filaments around its pathogen-containing vacuole (aka the inclusion). The formation of these actin structures is required for maintaining the integrity of the growing inclusion [25]. It is thus tempting to speculate that, in contrast to *Chlamydia*, which uses the RhoA-dependent actin cytoskeleton to stabilize its vacuolar niche, *S*. *flexneri* antagonizes RhoA recruitment to suppress actin-dependent stabilization of DMVs, thereby promoting DMV escape. However, while our results do not exclude a role for dynamic actin polymerization in the process of *S*. *flexneri* DMV escape, the dramatic difference we observed in RhoA recruitment on *ipgB1* DMVs compared to wild type DMVs did not translate into differences in polymerized actin around the DMV (S6 Fig). Therefore, we do not favor a model in which RhoA restricts DMV escape through its role in regulation of the actin cytoskeleton.

#### The membrane trafficking hypothesis

Membrane trafficking, which is becoming increasingly recognized as being regulated by Rho GTPases [36,37] is emerging as a key influencer of pathogen vacuolar stability in the field of host-pathogen interactions [38]. There are several examples that involve RhoA as a membrane-bound compartment stabilizer in this context. For instance, the formation of the *Coxiella*-containing vacuole (CCV) relies on endosomal/lysosomal fusion events that are required for delivering membrane to expand the CCV. The *Coxiella* type IV secretion system effector, CirA, recruits and stimulates the activity of RhoA, which regulates the size of CCV [24,26], possibly by incorporating membrane from favorable trafficking events. Similarly, the *Salmonella* effectors, SseJ and SseL, contribute to vacuole stability by rerouting the host lipid transporter OSBP1 to the *Salmonella*-containing vacuole (SCV)[39]. Interestingly, this stabilization process relies on RhoA through SseJ recruitment and activation on SCVs [40–42]. Thus, RhoA emerges as an important factor in the modulation of membrane trafficking events critical for the stability of pathogen-containing vacuoles. In the context of *S*. *flexneri* DMV escape, it is therefore tempting to speculate that IpgB1 antagonizes RhoA recruitment in order to block membrane trafficking events, that would otherwise lead to vacuole stabilization. Combined with the tension applied to the vacuole as a consequence of bacterial growth, antagonizing RhoA recruitment may thus lead to membrane rupture and promote *S*. *flexneri* DMV escape.

#### How does IpgB1 contribute to pathogenesis?

We provide the first demonstration in a relevant animal model that a *S*. *flexneri* mutant lacking the T3SS effector IpgB1 is attenuated *in vivo*. Consistent with our experiments in HT-29 cells, *ipgB1* was less invasive (fewer infection foci) and less efficient at cell-to-cell spread (smaller infection foci). Animals infected with *ipgB1* had attenuated symptoms of illness, including epithelial fenestration and bloody diarrhea. We have previously shown that infection with a non-invasive *mxiG* mutant [9] does not cause epithelial fenestration or diarrhea, demonstrating that T3SS-dependent invasion of epithelial cells is necessary for pathogenesis in our infant rabbit model [3]. However, invasion alone is not sufficient for causing symptoms as a mutant lacking *icsA*, which is equally as invasive as wild type but deficient in cell-to-cell spread, does not cause fenestration or bloody diarrhea [3]. The *ipgB1* mutant produced fewer foci than wild type, but unlike *mxiG* still retained some ability to invade. The *ipgB1* mutant also produced smaller foci than wild type bacteria, but unlike *icsA*, maintained some level of cell-to-cell spread. It is therefore likely that the attenuated symptoms of illness caused by the *ipgB1* mutant are the combined result of decreased epithelial invasion, leading to fewer potential sites of fenestration, and diminished cell-to-cell spread, resulting in decreased fenestration and less severe bloody diarrhea. These *in vivo* studies validated our findings in tissue culture and provide evidence that IpgB1 is required for severe *S*. *flexneri* pathogenesis.

## Material and methods

### Ethics statement

All experiments described in this study were reviewed and approved by the University of Virginia Institutional Biosafety Committee and the Institutional Animal Care and Use Committee (protocol #4161).

### Cell lines and bacterial strains

HT-29 cells (ATCC HTB-38) were cultured at 37°C with 5% CO_2_ in McCoy’s 5A medium (Gibco) supplemented with 10% heat-inactivated fetal bovine serum (FBS) (Invitrogen). The wild-type *Shigella flexneri* strain used in this study is serotype 2a 2457T [2]. The *ipgB1* and *ipgB2* strains of *S*. *flexneri* were generated by allelic exchange resulting in replacement of the open reading frame (ORF) in the *S*. *flexneri* large virulence plasmid by the coding region of a kanamycin or chloramphenicol resistance cassettes, respectively. The *ipgB1; ipgB2* double mutant was created by replacing the *ipgB1* ORF with a kanamycin resistance cassette and replacing the *ipgB2* ORF with a chloramphenicol resistance cassette. The *icsB; ipgB1* mutant was created by replacing the *icsB* ORF, *ipgB1* ORF, and the intervening region containing *ipgA (*chaperone for IcsB) with a kanamycin resistance cassette. The *ipgB1* mutant was complemented by expressing *ipgB1* from the arabinose-inducible pBAD promoter in vector pBAD18 (ATCC 87393). The catalytically dead allele of IpgB1 (E80A) [20] was generated by overlap PCR. Primers used for creating the *ipgB1* and *ipgB2* mutants and for cloning are listed in S1 Table.

### DNA constructs and cell transfection

The HT-29 cell line stably expressing yellow fluorescent protein (YFP) membrane markers was generated using the pMX-MbYFP vector [7]. RhoA siRNA Duplex 2-resistant RhoA was created by overlap PCR and cloned into the XhoI and NotI sites of the pMX_mCherry vector, resulting in the generation of a N-terminal mCherry fusion protein (mCherry-siResistant RhoA). The corresponding lentiviruses were generated in 293T cells co-transfected with the packaging constructs pCMVΔ8.2Δvpr (HIV helix packaging system) and pMD2.G (a vesicular stomatitis virus glycoprotein) as previously described [43].

### Bacterial infection

*S*. *flexneri* was grown overnight in LB broth at 30°C with agitation. The bacteria were diluted 1:100 and grown to exponential phase for approximately 3 hr at 37°C with agitation. Cells were infected with *S*. *flexneri* expressing CFP under the control of an isopropyl-β-d-thiogalactopyranoside (IPTG)-inducible promoter. Infection was initiated by centrifuging the plate at 1,000 rpm for 5 min, and internalization of the bacteria was allowed to proceed for 1 hr at 37°C before gentamicin (50 μg/ml) and IPTG (10 mM final concentration) were added to kill the extracellular bacteria and induce CFP expression, respectively. For EHT 1864 Rac1 inhibitor (Target Mol T6483) experiments, EHT 1864 or mock (H20) was added (final concentration 20μM) at 1 hr postinfection for the remainder of the infection. For time-lapse microscopy, imaging began 2 hr postinfection. For *S*. *flexneri* focus size analysis, infected cells were incubated at 37°C for 16 hr. For gentamicin protection assays, infected cells were lysed and lysates plated for CFU at 2 hr. For foci number enumeration, infected cells were incubated at 37°C for 8 hr.

### siRNA transfection

Cells were transfected by reverse transfection with four Dharmafect1 individual small interfering RNAs (siRNAs) (50 nM total final concentration) targeting Rac1, RhoA, or siRNA buffer alone (mock) 96 h in a 96-well plate format (Corning 3904). Knockdown efficiency was determined by Western blotting. Cells were infected or collected for western blot analysis on day four of knockdown.

### Western blotting

Cells were directly lysed in 2x Laemmli buffer with 10mM DTT and separated by SDS-PAGE. Proteins were transferred onto nitrocellulose membranes and the membranes were incubated for 1 hr at room temperature with gentle shaking in blocking buffer (5% skim milk in 1X TBS with 0.05% Tween). The membranes were probed with primary antibodies diluted in blocking buffer overnight at 4°C with shaking. Secondary antibodies (HRP-conjugated) were diluted in blocking buffer and incubated with membranes for 1 hr room temperature with gentle shaking. ECL Standard Western blotting detection reagents (Amersham) were used to detect HRP-conjugated secondary antibodies on a BioRad ChemiDoc imaging system. Western blot quantification was performed using Fiji.

### Antibodies

The following antibodies were used for western blot (WB) and/or immunofluorescence (IF): mouse monoclonal anti-RhoA (Santa Cruz sc-418; WB 1:200, IF 1:50), mouse monoclonal anti-Rac1 (Cytoskeleton ARC03; WB 1:500), mouse monoclonal anti-Rac1 (BD Biosciences 610650; IF 1:50), rabbit polyclonal anti-Actin (Sigma WB 1:10,000), HRP-conjugated goat anti-rabbit IgG (Jackson WB 1:10,000), HRP-conjugated goat anti-mouse IgG (Jackson WB 1:10,000), AlexaFluor 594-conjugated goat anti-mouse IgG (Molecular Probes IF 1:1000). For actin visualization, AlexaFluor 594-conjugated Phalloidin (Invitrogen A12381 IF 1:1000) was used.

### Immunofluorescence

Monolayers of HT-29 cells were grown on collagen-coated (Sigma C3867, 1:100) glass coverslips and infected with *S*. *flexneri* for 5 hr, then fixed with 4% paraformaldehyde in 1x PBS for 20 minutes at room temperature and washed three times with 1x PBS. Coverslips were incubated with primary antibody diluted in 0.1% triton X-100 in 1x PBS overnight at 4°C in humidity chamber. Coverslips were then washed with 1x PBS and incubated in secondary antibody diluted in 0.1% triton X-100 in 1x PBS for 1 hr at room temperature. For phalloidin staining, coverslips were incubated with phalloidin conjugated to AlexaFluor 594 (Invitrogen A12381) diluted in 0.1% triton X-100 in 1x PBS for 2 hr at room temperature. Coverslips were washed with PBS and then mounted with DABCO. Confocal imaging was performed using an Andor iXon ULTRA 888BV EMCCD camera and a Yokogawa CSU-W1 confocal scanner unit attached to a Leica DMi8 microscope. Z slices were captured in 1 μm increments spanning the entire cell.

### Size of infection foci

The size of infection foci formed in HT-29 cells and infected with CFP-expressing *S*. *flexneri* strains was determined in a 96-well plate format. After fixation with 4% paraformaldehyde for 20 minutes at room temperature, the plates were imaged using the ImageXpress Micro imaging system (Molecular Devices). Margins of individual foci were defined manually, and image analysis for foci size (area) was performed using the region measurement function in the ImageXpress imaging software (Molecular Devices) as previously described [8]. Unless otherwise specified, image analyses were conducted on at least 50 infection foci in each biological replicate (n) and for at least three biological replicates (N).

### Live imaging

Bacterial dissemination was monitored using time-lapse confocal microscopy. Plasma membrane YFP-expressing HT-29 cells were grown in collagen-coated (Sigma C3867, 1:100) eight-well chambers (Lab-Tek II (Thermo Fisher Scientific catalog no. 155409) at 37°C in 5% CO_2_. Cells were infected with CFP-expressing *S*. *flexneri* strains and centrifuged at 800 rpm for 4 min. Bacteria were then allowed to invade for 1 hr before gentamicin (50 μg/ml) and IPTG (10 mM final concentration) were added to kill the extracellular bacteria and induce CFP expression, respectively. Starting 2 hrs postinfection, cells were imaged with a Leica DMI 8 spinning-disc confocal microscope driven by the iQ software (Andor). Z-stacks were captured every 2 min for 6 hrs. The corresponding movies were generated with Imaris software (Bitplane). For tracking analysis, protrusions were defined as plasma membrane extensions that formed as a result of bacteria reaching the cell cortex and projecting into adjacent cells. Vacuole-like protrusions (VLPs) were defined as an intermediate compartment between protrusions and vacuoles, characterized by a continuous membrane lining around the bacteria and a membranous tether. Double-membrane vacuoles (DMVs) were defined as membrane-bound compartments that derived from VLPs after resolution of the membranous tether. As opposed to VLPs, DMVs were therefore no longer connected to the primary infected cell. Free (cytosolic) bacteria were defined as bacteria that were previously observed in vacuoles but were no longer surrounded by a continuous lining of the plasma membrane and have regained actin-based motility.

### *In vivo S*. *flexneri* infection

Animal studies were conducted using our previously described infant rabbit model of *S*.*flexneri* infection [3]. Newborn New Zealand White rabbits were isolated after birth and kept at 30 °C, with regular feedings from tranquilized does. Infant rabbits were infected at 10–15 days old. *S*. *flexneri* was grown overnight at 37 °C on a rotating wheel in 5 ml tryptic soy broth (TSB) per animal. Prior to infection, the bacteria were pelleted and resuspended in PBS. Kits were anesthetized with 5% isoflurane and rectally inoculated with 200 μl of bacterial suspension (~10^9^ CFUs per animal). Animals were monitored twice daily for clinical signs of illness.

### Histology

For clinical scoring of infection (blood, diarrhea, and fenestration), animals were euthanized by CO_2_ inhalation and the distal colon was harvested at 24 hrs postinfection. Scoring of blood and diarrhea were performed blindly at the time of tissue harvest based on color and wetness of hind limb fur stain. Blood and dysentery scores were determined based on the presence and severity of blood and diarrhea on the hind limb fur and were scored as (1) genitals only, (2) genitals and belly and (3) genitals, belly and legs. For determination of foci number (invasion) and foci size (cell-to-cell spread) *in vivo*, colons were harvested at 4 hr and 8 hr postinfection, respectively. The distal colons were rinsed in PBS and flushed with modified Bouin’s fixative, then cut open longitudinally and displayed in cassettes as swiss-rolls. Cassettes were then immersed in neutral-buffered formalin. The tissue was preserved in 70% EtOH before loading onto a tissue processor for dehydration and paraffin infiltration. After manual embedding into a paraffin block, paraffin sections were cut at 5 μm on a Leica microtome. Paraffin sections were stained with hematoxylin and eosin and imaged using ScanScope Slide Scanner (Leica Biosystems). Epithelial fenestration was measured along entire colon using the Aperio software and % fenestration (length of colon with fenestrated epithelium/total length of colon x 100) was calculated for each colon.

### Immunofluorescence on colon sections

Paraffin sections on slides were deparaffinized and re-hydrated as previously described [3]. Antigen retrieval in pre-heated citric acid based buffer (1:100 in H2O, Vector Laboratories, H-3300) was performed in a pressure cooker (Instant Pot) for 1 min on the high pressure setting, followed by a “quick release.” Slides were rinsed three times with PBS, then permeabilized in 0.1% Triton in PBS for 10 minutes at room temperature and blocked in 5% bovine serum albumin and 2% normal goat serum in PBS for 1 hr at room temperature. Primary antibodies (mouse anti-E-cadherin, BD Biosciences 610181, 1:100; rabbit anti-*Shigella*, ViroStat 0901, 1:100) were diluted in blocking buffer and incubated overnight at 4 °C in humidity chamber. Slides were washed three times with PBS, the secondary antibodies (goat anti-mouse IgG Alexa Fluor 514, Thermo Fisher, 1:500; goat anti-rabbit IgG Alexa Fluor Pacific Blue, Thermo Fisher, 1:500) were diluted in blocking buffer and incubated for 2 hrs at room temperature. Coverslips were mounted using 1 drop of ProLong Gold Antifade (Thermo Fisher) per slide and imaged with a Nikon TE2000 microscope equipped for automated multi-color imaging including motorized stage and filter wheels, a Hamamatsu Orca ER Digital CCD Camera and piezo-driven 10x and 60x objectives. The images were processed with the MetaMorph software (Molecular Devices, Inc.). For foci number analysis, the number of foci per colon was enumerated along the entire distal colon for each animal. Foci sizes were measured using the region measurement tool of the MetaMorph software as described above.

## Supporting information

S1 Fig IpgB2 is not required for invasion or cell-to-cell spread in HT-29 cells. (A) Intracellular CFUs from HT-29 cells at 2 hr postinfection normalized to inoculum. Three independent biological replicates were performed, each containing three technical replicates. Each point represents one technical replicate and the grey bars represent the mean number of CFU. (B) Number of infection foci formed at 8 hr postinfection normalized to inoculum. Three independent biological replicates were performed, each containing five technical replicates. Each point represents one technical replicate and the grey bars represent the mean number of foci. (C) Quantification of foci size (area) in arbitrary units at 16 hr postinfection. Three independent biological replicates were performed, each containing at least 50 foci. Each point represents one focus and the grey bars represent the mean foci size. Error bars represent the standard deviations; unpaired two-tailed t-test; ns, not significant; ****, p<0.0001.
(TIF)

S2 Fig The phenotypes of *ipgB1/ ipgB2* and *icsB/ ipgB1* double mutants. Quantification of foci size (area) in arbitrary units at 16 hr postinfection for either (A) *ipgB1/ipgB2* or (B) *icsB/ipgB1* double mutants. Three-six independent biological replicates were performed, each containing at least 50 foci. Each point represents one focus and the grey bars represent the mean foci size. Error bars represent the standard deviations; one-way ANOVA was performed with Dunnett’s multiple comparisons test; ns, not significant; ***, p<0.001; ****, p<0.0001.
(TIF)

S3 Fig The protrusion branching phenotype. (A-B) Still images from (A) non-branching or (B) branching cell-to-cell spread example. Images correspond to S1 and S2 Movie, respectively. White arrowheads indicate which bacteria to follow as examples. The time in minutes corresponding to each image is shown in bottom right corner. Each example begins at 2 min, the first frame at which the bacterium is in a protrusion. At 0 min, the bacterium is in the primary infected cell. Scale bar, 2 μm. (C) Graph depicting the percentage of wild type or *ipgB1* bacteria that display protrusion branching during spreading. Each dot represents percentage from one biological replicate and grey bars show average of 4 biological replicates. Error bar indicates standard deviation. (D-F) Graphs depicting (D) time spent in membrane compartments (protrusions, VLPs, DMVs), (E) time spent in protrusions and VLPs, or (F) time spent in DMVs by *ipgB1* bacteria displaying non-branching or branching protrusions. Each dot represents one bacterium and the grey bars represent the average of all tracked bacteria per category. Error bars indicate standard deviations. Unpaired two-tailed t-test; ns, not significant. (G) Graphs showing the proportions of fates of all tracked *ipgB1* bacteria from 4 biological replicates.
(TIF)

S4 Fig Rac1 depletion decreases DMV escape. (A and B) Representative tracking analysis of CFP-expressing wild type bacteria in (A) mock-treated or (B) Rac1-depleted HT-29 cells expressing plasma membrane-targeted YFP. Each bar represents the tracking of a single bacterium over 180 minutes. Thicker bars indicate instances when bacteria divided and branched during spreading. At least 30 bacteria were tracked for condition per biological replicate. (C) Graphs showing the proportions of fates of tracked bacteria from three biological replicates. Standard deviations of the means are indicated. Two-way ANOVA with Sidak’s multiple comparisons was performed; **, p<0.005; ***, p<0.001; ****, p<0.0001. Time spent in (D) protrusions, (E) VLPs, or (F) DMVs is shown for all tracked bacteria. Each dot represents a single bacterium and the grey bars show the mean of all tracked bacteria from three biological replicates. Error bars indicate standard deviation. Unpaired two-tailed t-tests were performed; ns, not significant.
(TIF)

S5 Fig IpgB1 modulates levels of Rac1 and RhoA DMVs. (A) Quantification of the percentage of RhoA-positive DMVs per foci in mock-treated or Rac1 duplex 3-depleted HT-29 cells infected with wild type or *ipgB1* bacteria. Each dot represents the percent RhoA-positive DMVs in a focus and the grey bars show the mean percentage of RhoA-positive DMVs from all foci measured in three biological replicates. (B) Quantification of the percentage of Rac1-positive DMVs in mock-treated or RhoA duplex 2-depleted HT-29 cells infected with wild type or *ipgB1* bacteria. Each dot represents the percent Rac1-positive DMVs in a focus and the grey bars show the mean percentage of Rac1-positive DMVs from all foci measured in three biological replicates. At least ten foci per condition were analyzed in each biological replicate. Error bars indicate standard deviation. One-way ANOVA with Tukey’s multiple comparisons; ns, not significant; ***, p<0.001; ****, p<0.0001.
(TIF)

S6 Fig IpgB1 does not affect actin accumulation around DMVs. (A and B) Confocal images of HT-29 cells stably expressing plasma membrane-targeted YFP and infected for 5 hr with CFP-expressing *S*. *flexneri* and stained using AlexFluor 594-conjugated phalloidin. Merged images are shown on the left, single YFP channel in middle, and single mCherry channel on the right. Scale bar, 2 μm. (A) Representative example of a DMVs that is positive for actin recruitment. (B) Representative example of a DMV that is negative for actin recruitment. (C) Quantification of the percentage of wild type or *ipgB1* DMVs per focus associated with actin at 5 hr postinfection. Each dot represents the percent positive DMVs in a focus and the grey bars show the mean percentage of actin-positive DMVs from all foci measured in three biological replicates. At least twelve foci per condition were analyzed in each biological replicate. Error bars indicate standard deviation. Unpaired two-tailed t-test; ns, not significant.
(TIF)

S1 Table Primers pairs (name and sequence) and corresponding templates used in this study. (XLSX)

S2 Table Rho family siRNA screen. (Sheet 1) Raw data from siRNA screen. Column B indicates the position of the well within the 384 screening plate and the corresponding gene name is found in column C. “#N/A” indicates wells containing siRNA buffer alone and were used to calculate average foci size for mock condition. Blank wells in column C indicate wells located on the edge of the plate and were thus not included in the analysis due to potential edge effect. For each replicate, wild type and *ipgB1* averages and standard deviations are calculated as well as lower and upper cutoff scores (1.5 standard deviations below or above the average, respectively). The median foci size per well is shown as well as the corresponding z-score. Wells that fall below lower cutoff or above upper cutoff are highlighted in green and red, respectively. (Sheet 2) Overview of screening data. Each gene name is displayed in column C. The “hit” criteria of how many duplexes were found (out of 4) per replicate and the number of replicates that this result must repeat (out of 4) are indicated in red and orange text, respectively. For wild type and *ipgB1*, the # of duplexes that resulted in decreased spreading below lower cutoff or increased spreading above upper cutoff for each replicate are shown. Columns A and B indicate the number of replicates in which the “hit” was found. Values for “hits” that decreased or increased spreading are highlighted in green and red, respectively.
(XLSX)

S1 Movie Example of non-branching cell-to-cell spread. Plasma membrane-YFP expressing HT-29 infected with wild type *S*.*flexneri* and imaged every 2 minutes for 6 hours. The corresponding movie shows the canonical progression of cell-to-cell spread.
(MP4)

S2 Movie Example of branching cell-to-cell spread. Plasma membrane-YFP expressing HT-29 infected with *ipgB1 S*.*flexneri* and imaged every 2 minutes for 6 hours. The corresponding movie shows the noncanonical, branching of the during progression of cell-to-cell spread.
(MP4)
