# Supplementary figures and images for: The type three secretion system effector protein IpgB1 promotes Shigella flexneri cell-to-cell spread through double-membrane vacuole escape
Source: PLoS Pathog. 2022 Feb 24;18(2):e1010380. doi: 10.1371/journal.ppat.1010380 (PMC8903249; doi:10.1371/journal.ppat.1010380)

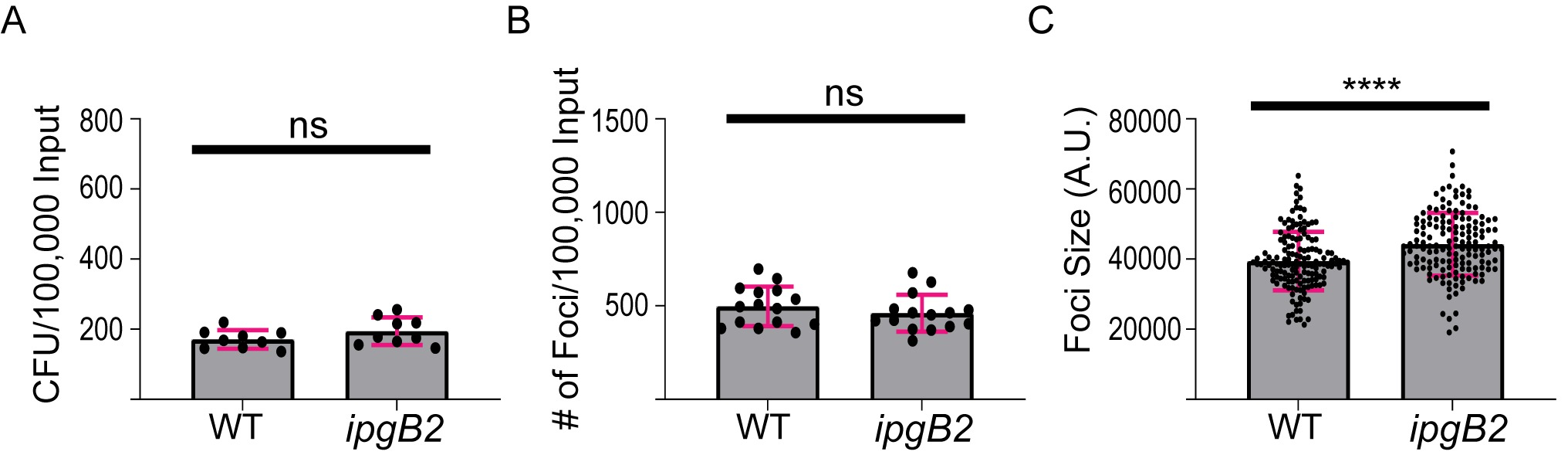

Supplement: S1 Fig — (A) Intracellular CFUs from HT-29 cells at 2 hr postinfection normalized to inoculum. Three independent biological replicates were performed, each containing three technical replicates. Each point represents one technical replicate and the grey bars represent the mean number of CFU. (B) Number of infection foci formed at 8 hr postinfection normalized to inoculum. Three independent biological replicates were performed, each containing five technical replicates. Each point represents one technical replicate and the grey bars represent the mean number of foci. (C) Quantification of foci size (area) in arbitrary units at 16 hr postinfection. Three independent biological replicates were performed, each containing at least 50 foci. Each point represents one focus and the grey bars represent the mean foci size. Error bars represent the standard deviations; unpaired two-tailed t-test; ns, not significant; ****, p<0.0001. (TIF) [file ppat.1010380.s001.tif]

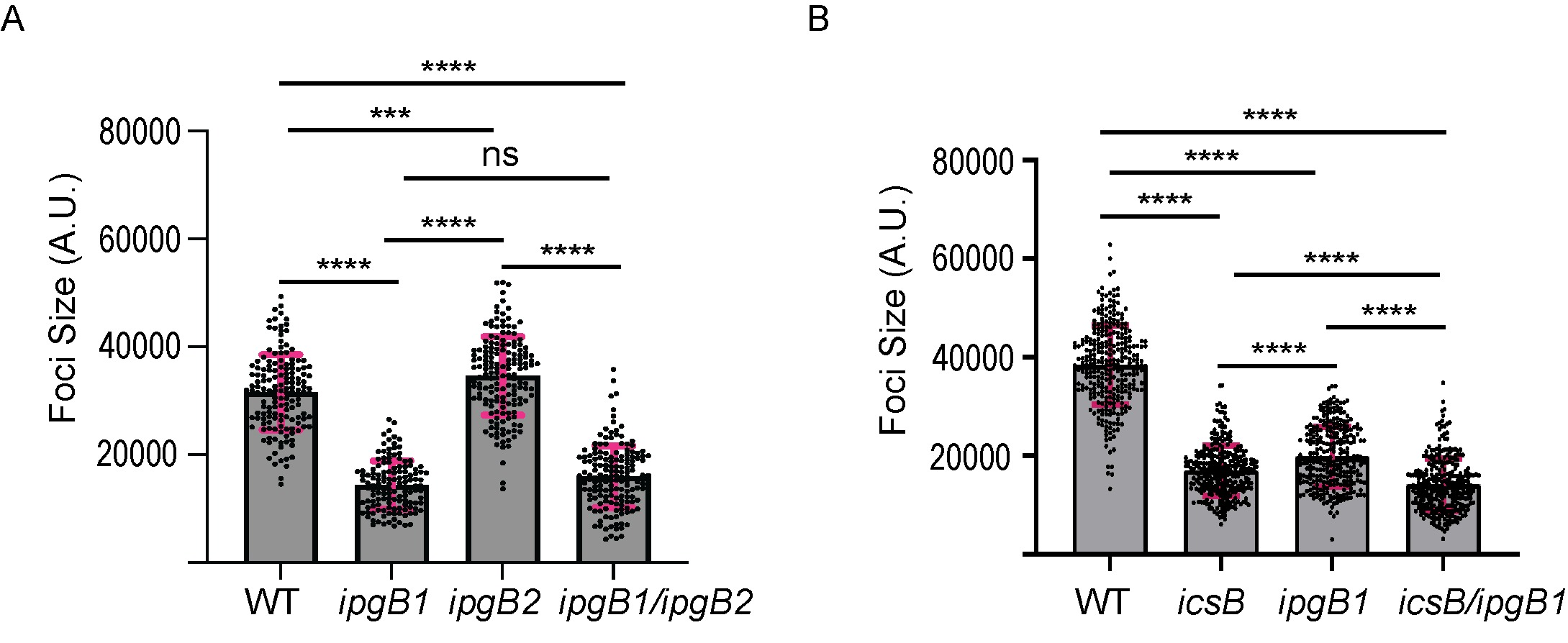

Supplement: S2 Fig — Quantification of foci size (area) in arbitrary units at 16 hr postinfection for either (A) ipgB1/ipgB2 or (B) icsB/ipgB1 double mutants. Three-six independent biological replicates were performed, each containing at least 50 foci. Each point represents one focus and the grey bars represent the mean foci size. Error bars represent the standard deviations; one-way ANOVA was performed with Dunnett’s multiple comparisons test; ns, not significant; ***, p<0.001; ****, p<0.0001. (TIF) [file ppat.1010380.s002.tif]

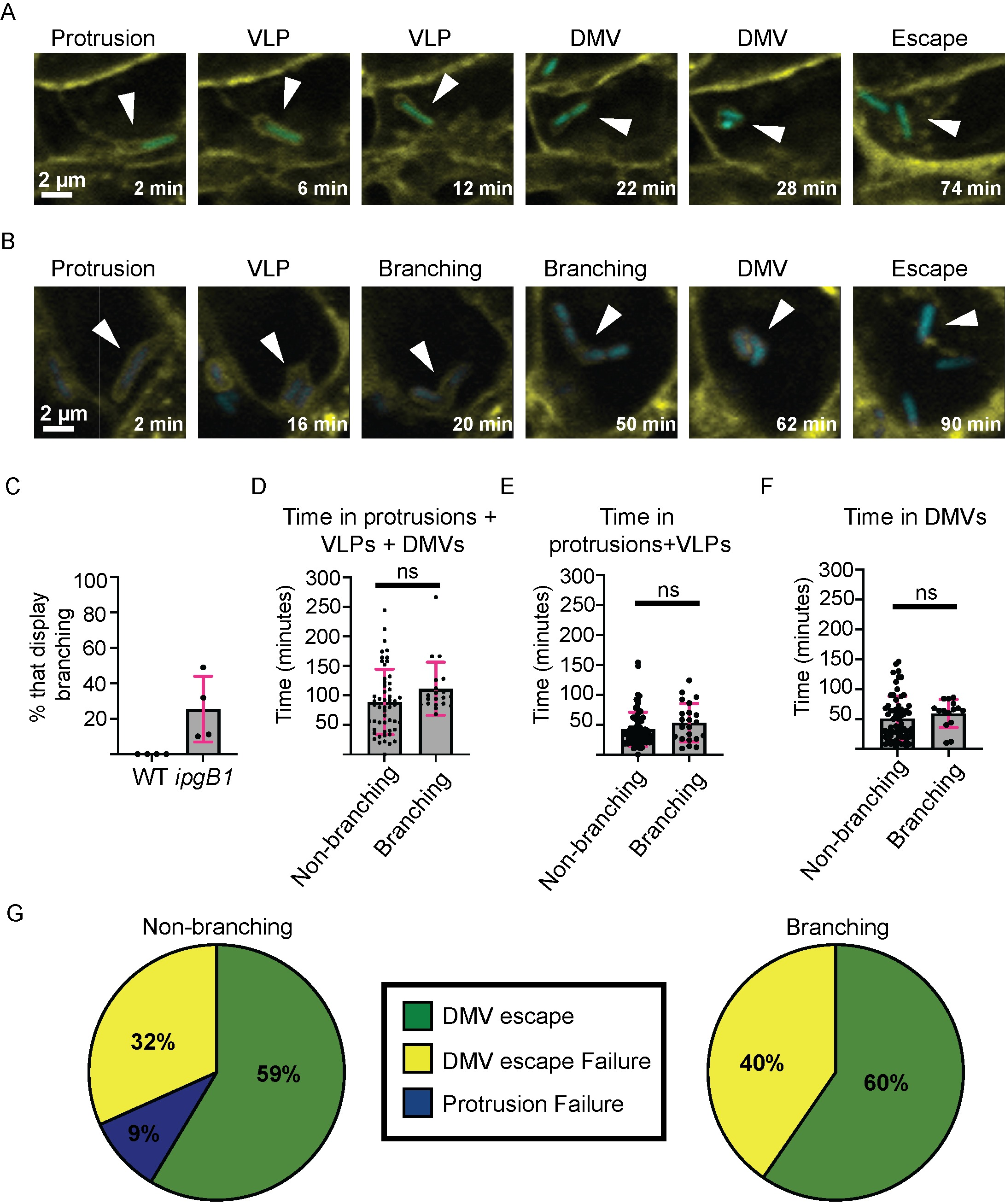

Supplement: S3 Fig — (A-B) Still images from (A) non-branching or (B) branching cell-to-cell spread example. Images correspond to S1 and S2 Movie, respectively. White arrowheads indicate which bacteria to follow as examples. The time in minutes corresponding to each image is shown in bottom right corner. Each example begins at 2 min, the first frame at which the bacterium is in a protrusion. At 0 min, the bacterium is in the primary infected cell. Scale bar, 2 μm. (C) Graph depicting the percentage of wild type or ipgB1 bacteria that display protrusion branching during spreading. Each dot represents percentage from one biological replicate and grey bars show average of 4 biological replicates. Error bar indicates standard deviation. (D-F) Graphs depicting (D) time spent in membrane compartments (protrusions, VLPs, DMVs), (E) time spent in protrusions and VLPs, or (F) time spent in DMVs by ipgB1 bacteria displaying non-branching or branching protrusions. Each dot represents one bacterium and the grey bars represent the average of all tracked bacteria per category. Error bars indicate standard deviations. Unpaired two-tailed t-test; ns, not significant. (G) Graphs showing the proportions of fates of all tracked ipgB1 bacteria from 4 biological replicates. (TIF) [file ppat.1010380.s003.tif]

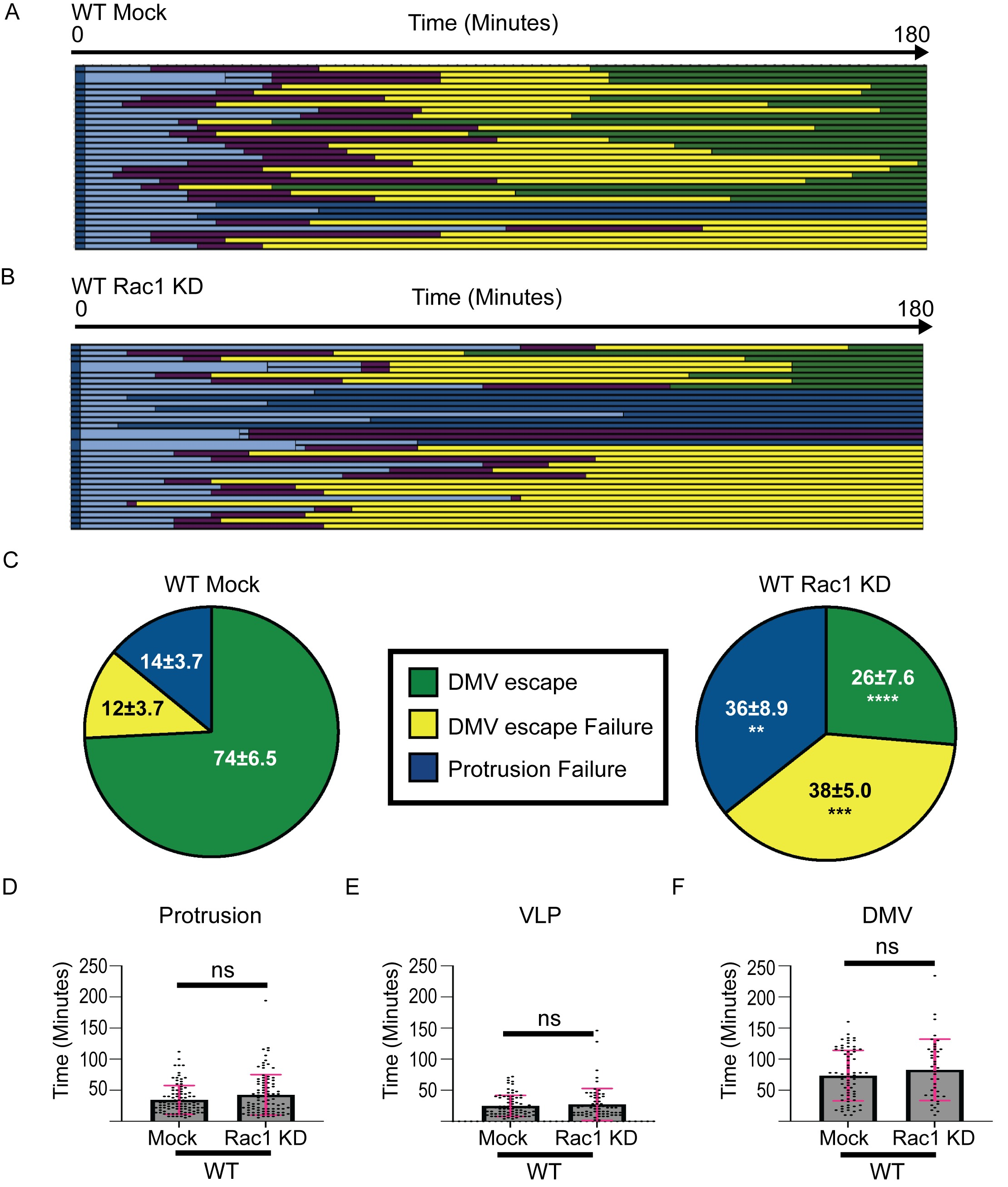

Supplement: S4 Fig — (A and B) Representative tracking analysis of CFP-expressing wild type bacteria in (A) mock-treated or (B) Rac1-depleted HT-29 cells expressing plasma membrane-targeted YFP. Each bar represents the tracking of a single bacterium over 180 minutes. Thicker bars indicate instances when bacteria divided and branched during spreading. At least 30 bacteria were tracked for condition per biological replicate. (C) Graphs showing the proportions of fates of tracked bacteria from three biological replicates. Standard deviations of the means are indicated. Two-way ANOVA with Sidak’s multiple comparisons was performed; **, p<0.005; ***, p<0.001; ****, p<0.0001. Time spent in (D) protrusions, (E) VLPs, or (F) DMVs is shown for all tracked bacteria. Each dot represents a single bacterium and the grey bars show the mean of all tracked bacteria from three biological replicates. Error bars indicate standard deviation. Unpaired two-tailed t-tests were performed; ns, not significant. (TIF) [file ppat.1010380.s004.tif]

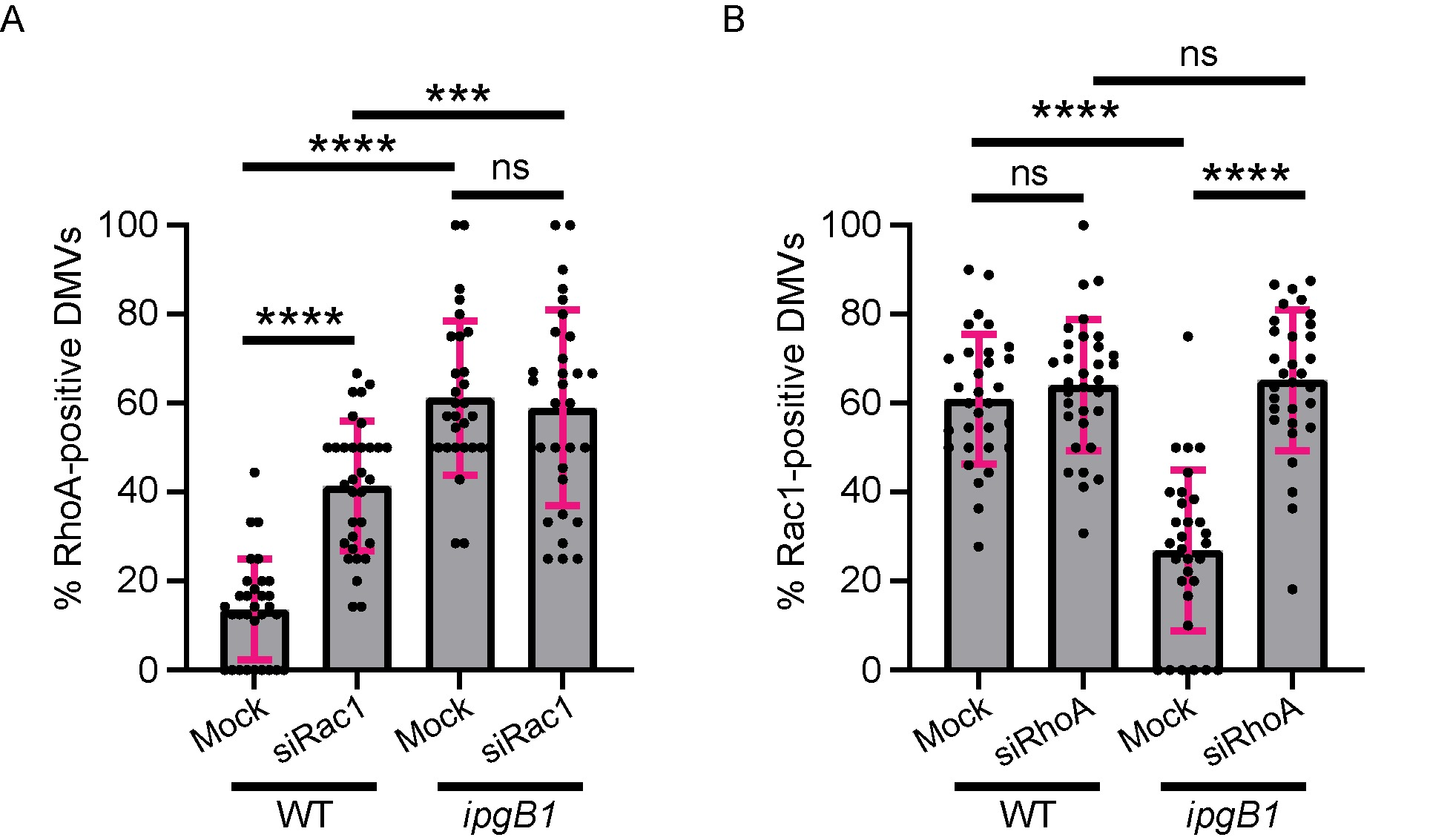

Supplement: S5 Fig — (A) Quantification of the percentage of RhoA-positive DMVs per foci in mock-treated or Rac1 duplex 3-depleted HT-29 cells infected with wild type or ipgB1 bacteria. Each dot represents the percent RhoA-positive DMVs in a focus and the grey bars show the mean percentage of RhoA-positive DMVs from all foci measured in three biological replicates. (B) Quantification of the percentage of Rac1-positive DMVs in mock-treated or RhoA duplex 2-depleted HT-29 cells infected with wild type or ipgB1 bacteria. Each dot represents the percent Rac1-positive DMVs in a focus and the grey bars show the mean percentage of Rac1-positive DMVs from all foci measured in three biological replicates. At least ten foci per condition were analyzed in each biological replicate. Error bars indicate standard deviation. One-way ANOVA with Tukey’s multiple comparisons; ns, not significant; ***, p<0.001; ****, p<0.0001. (TIF) [file ppat.1010380.s005.tif]

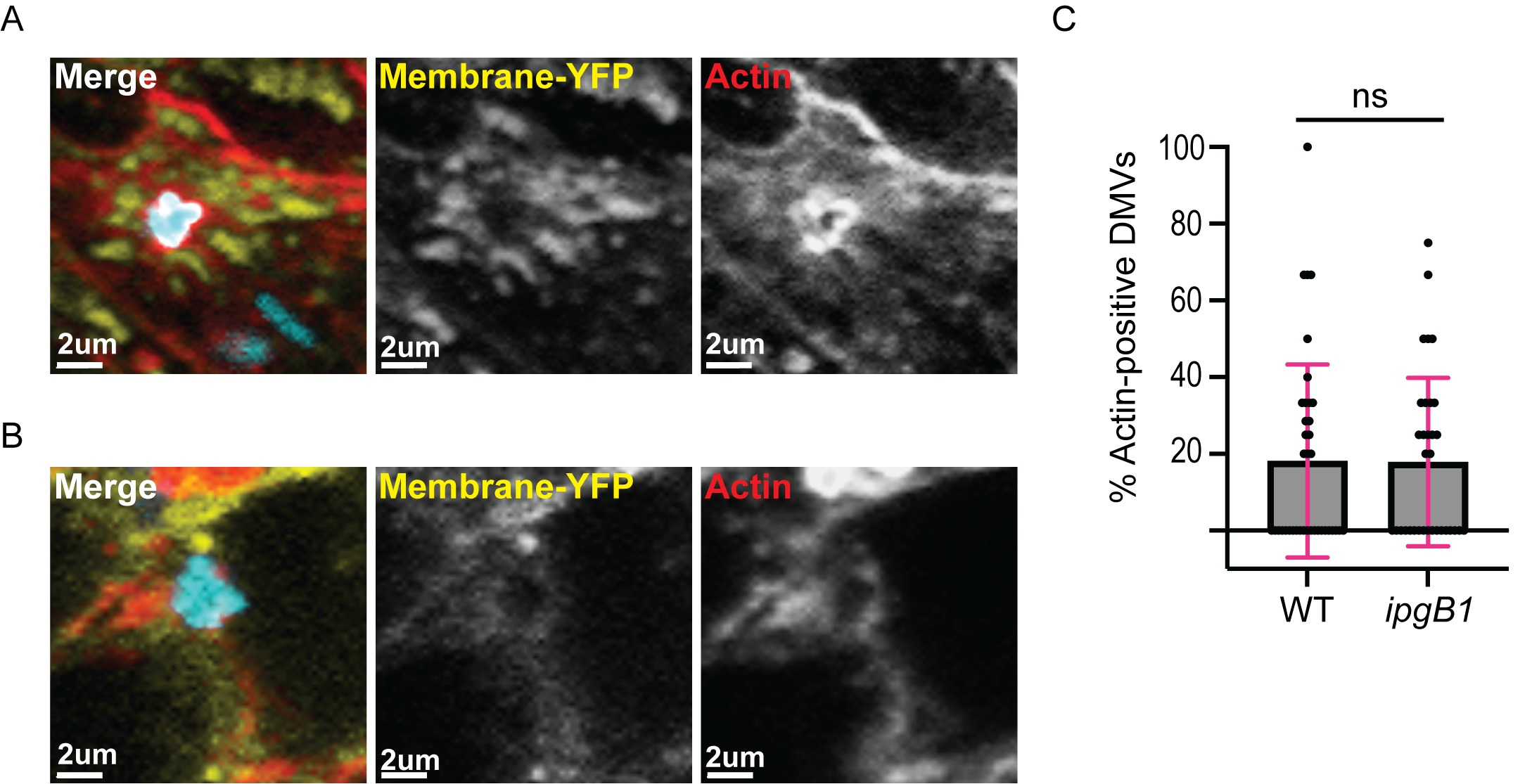

Supplement: S6 Fig — (A and B) Confocal images of HT-29 cells stably expressing plasma membrane-targeted YFP and infected for 5 hr with CFP-expressing S. flexneri and stained using AlexFluor 594-conjugated phalloidin. Merged images are shown on the left, single YFP channel in middle, and single mCherry channel on the right. Scale bar, 2 μm. (A) Representative example of a DMVs that is positive for actin recruitment. (B) Representative example of a DMV that is negative for actin recruitment. (C) Quantification of the percentage of wild type or ipgB1 DMVs per focus associated with actin at 5 hr postinfection. Each dot represents the percent positive DMVs in a focus and the grey bars show the mean percentage of actin-positive DMVs from all foci measured in three biological replicates. At least twelve foci per condition were analyzed in each biological replicate. Error bars indicate standard deviation. Unpaired two-tailed t-test; ns, not significant. (TIF) [file ppat.1010380.s006.tif]
